# Development of Decision Forest Models for Prediction of Drug-Induced Liver Injury in Humans Using A Large Set of FDA-approved Drugs

**DOI:** 10.1038/s41598-017-17701-7

**Published:** 2017-12-11

**Authors:** Huixiao Hong, Shraddha Thakkar, Minjun Chen, Weida Tong

**Affiliations:** Division of Bioinformatics and Biostatistics, National Center for Toxicological Research, US Food and Drug Administration, Jefferson, Arkansas 72079 USA

## Abstract

Drug-induced liver injury (DILI) presents a significant challenge to drug development and regulatory science. The FDA’s Liver Toxicity Knowledge Base (LTKB) evaluated >1000 drugs for their likelihood of causing DILI in humans, of which >700 drugs were classified into three categories (most-DILI, less-DILI, and no-DILI). Based on this dataset, we developed and compared 2-class and 3-class DILI prediction models using the machine learning algorithm of Decision Forest (DF) with Mold2 structural descriptors. The models were evaluated through 1000 iterations of 5-fold cross-validations, 1000 bootstrapping validations and 1000 permutation tests (that assessed the chance correlation). Furthermore, prediction confidence analysis was conducted, which provides an additional parameter for proper interpretation of prediction results. We revealed that the 3-class model not only had a higher resolution to estimate DILI risk but also showed an improved capability to differentiate most-DILI drugs from no-DILI drugs in comparison with the 2-class DILI model. We demonstrated the utility of the models for drug ingredients with warnings very recently issued by the FDA. Moreover, we identified informative molecular features important for assessing DILI risk. Our results suggested that the 3-class model presents a better option than the binary model (which most publications are focused on) for drug safety evaluation.

## Introduction

Predicting drug-induced liver injury (DILI) is a challenge for drug developers and regulators^1^. Despite many efforts to eliminate hepatotoxic drugs before they are tested in humans, hepatotoxic drugs often escape preclinical toxicity testing and are not identified as hepatotoxic until in a later stage of drug development and sometimes even after the approval^2^. Therefore, there is an unmet need for predicting human DILI risk during preclinical testing. A variety of approaches has been evaluated to estimate human DILI risks including *in silico* ^3–9^, *in vitro* ^10–13^, and *in vivo* ^14–16^. Although *in vitro* and *in vivo* studies and clinical investigations improved our understanding of human DILI risk of drug products, human DILI risk prediction remains to be a challenge in drug development, regulatory science, and clinical practices.

As assessing DILI risk using *in vitro* and *in vivo* experiments is time-consuming and expensive, *in silico* methods are attractive to scientists for prediction of human DILI risk due to cost effectiveness and easy implementation^17,18^. Therefore, many *in silico* quantitative structure-activity relationship (QSAR) models have been developed to predict DILI risk^3–9^. However, most of the reported *in silico* models have limitations^19^. Firstly, they were trained to predict drugs in two types: DILI and no-DILI drugs. Secondly, they were not thoroughly validated. For example, only one holdout validation may not result in a robust estimation of the model performance^7^. Lastly, a large set of drugs with a consistent DILI classification scheme is essential for the success of DILI prediction models; but such set of drugs were not available before.

A big number of drugs with reliable DILI classifications are critical for the development of robust and accurate *in silico* DILI prediction models^20^. A reliable DILI classification method should incorporate the frequency, causality, and severity of DILI^19^. The FDA-approved drug labeling is the authoritative document which comprehensively considers these factors to summarize drug safety information from clinical trials, post-marketing surveillance, and literature publications^21^. This set of drugs was recommended as the standard list for developing DILI predictive models^22,23^. Recently, we have further refined DILI classification for 1036 drugs by combining their FDA drug labeling information and causality assessment reports in the literature. This refined approach classified a large set of drugs into four classes: most-DILI, less-DILI, no-DILI, and ambiguous DILI^24^. This set of drugs with DILI classifications enables development of DILI risk prediction models that overcome some of the mentioned limitations.

In this study, we developed DILI prediction models using a pattern recognition algorithm Decision Forest (DF)^25,26^ based on this largest set of drugs (named DILIrank)^24^. The DF models were validated using 1,000 iterations of cross-validations and 1000 bootstrapping to reach statistically robust estimations of model performance. In addition to 2-class prediction models as reported in the literature, we also developed 3-class prediction models to further differentiate drugs with less-DILI from ones with most-DILI. The results showed that our models had better performance than the models published by our group and other teams. Our models were tested to ingredients of the drugs with warnings of the risk of serious liver injury that were recently issued by the FDA. Our models could be helpful in identification of potential DILI drugs during preclinical development and thus would be eventually beneficial in reducing hepatotoxicity related attrition.

## Results

### Models developed

**Figure 1:**
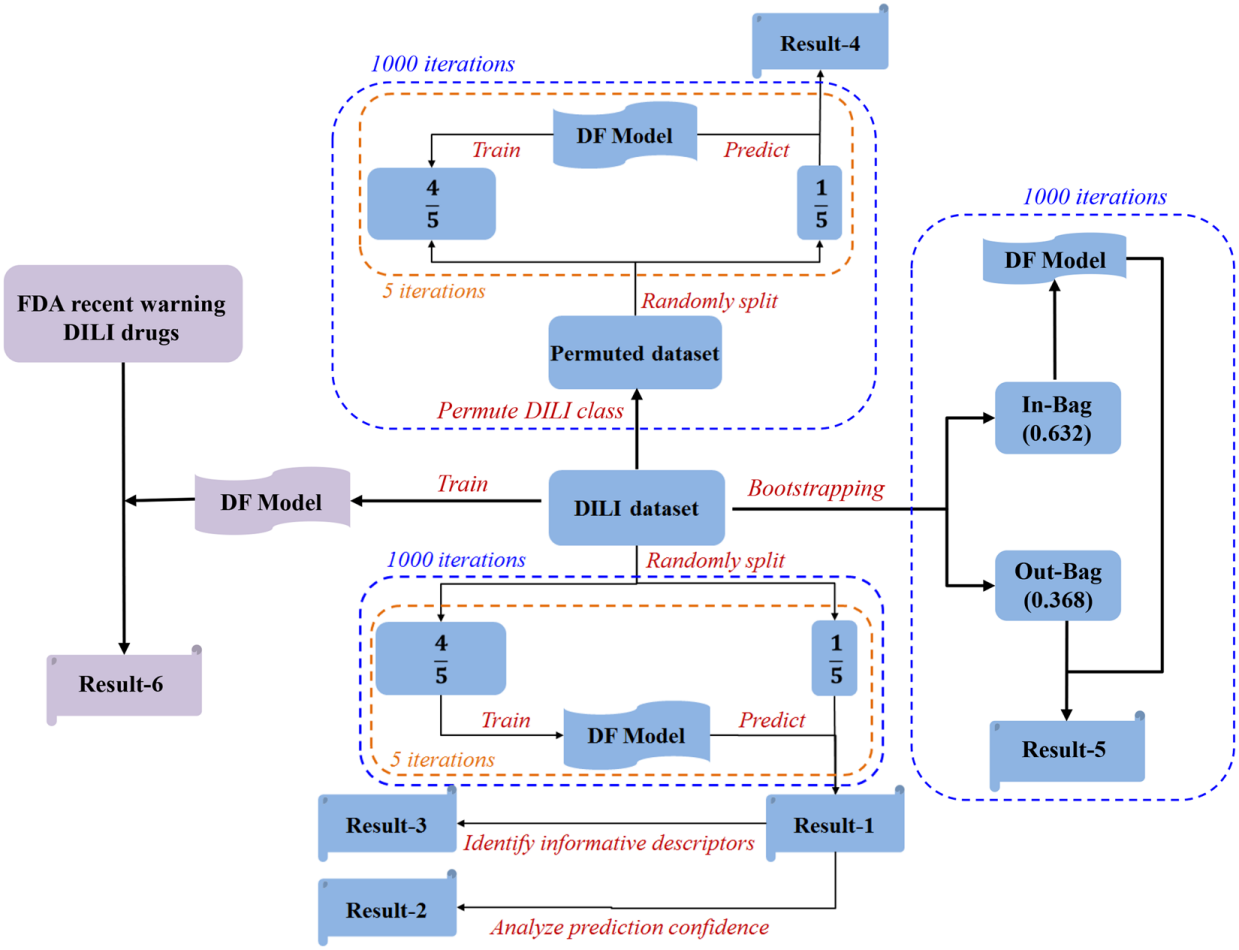
Study design and results yield. Actual dataset indicates the 2-class dataset of 451 drugs or the 3-class dataset of 721 drugs extracted from DILIrank data. Result-1 held the predictions from 5-fold cross-validations. Result-2 contained the prediction confidence values calculated for each prediction from the cross-validations. The frequency data for Mold2 descriptors used in the DILI prediction models were stored as Results-3. Result-4 was the collection of predictions from the random models developed from the permutated datasets. Result-5 depicted the results yielded from bootstrapping (strategy A) validations. Result-6 indicated proactive DILI predictions for the five drug ingredients of three drugs.

To develop reliable models for prediction of human DILI risk, we used a set of 721 drugs, from DILIrank^24^, that were classified into three classes with different human DILI risk (most-, less-, and no-DILI) based on FDA drug labeling. The study design of model development and validations was illustrated in Fig. 1. Of the 721 drugs, 268, 183, and 270 were no-, most-, and less-DILI drugs, respectively. Names, CASRN, SMILES codes along with the DILI classification of the 721 drugs were given in Supplementary Table S1. The 721 drugs were used for 3-class DF model development and validation as illustrated in Fig. 1.

DILI prediction models reported in the literature are 2-class prediction models: drugs were predicted to be DILI or no-DILI drugs. Therefore, the 268 no-DILI- and 183 most-DILI drugs were used for 2-class prediction model development and validation using the same procedures used for the 3-class models as illustrated in Fig. 1. An actual dataset (721 for 3-class or 451 for 2-class) was used for 1000 iterations of 5-fold cross-validations and bootstrapping validations. The results were used for the estimation of performance of the DF prediction models, assessment of prediction confidence, and identification of informative chemical descriptors. The actual dataset was then used in 1000 runs of permutation tests. The permutation tests were compared with the 5-fold cross-validations and bootstrapping to estimate if the DF models developed from the actual dataset could be obtained solely by chance. The models developed were tested on the drugs for which FDA recently issued DILI warnings.

### Cross-validations

**Figure 2:**
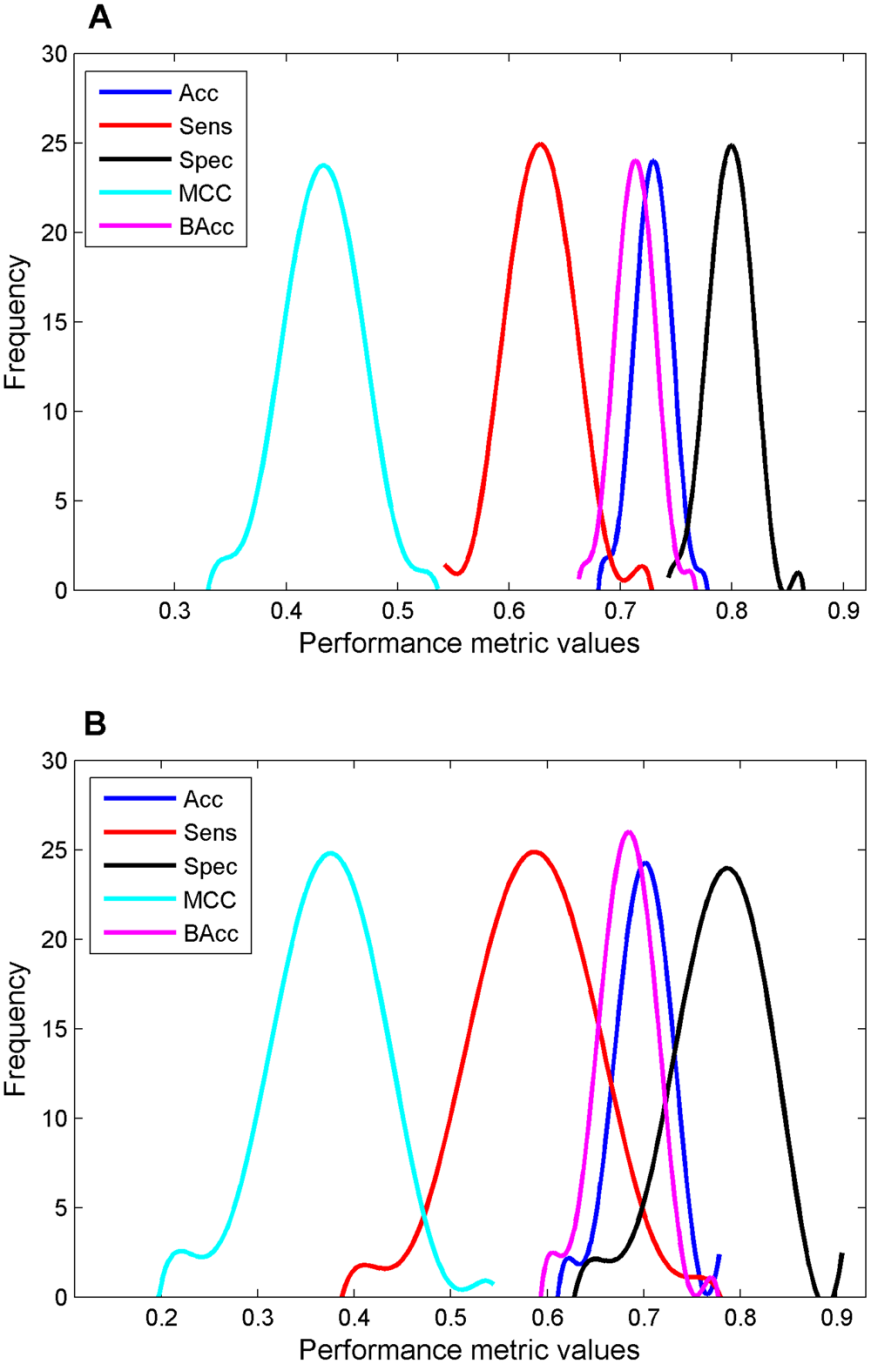
DILI risk prediction performance of the 5-fold cross-validations (**A**) and strategy A bootstrapping (**B**) on the 2-class dataset. Distributions of DILI prediction accuracy (blue), sensitivity (red), specificity (black), MCC (cyan) and balanced accuracy (magenta) of the 1000 iterations of 5-fold cross-validations and of the 1000 bootstrapping were plotted. Performance metrics values were given at the x-axis and the y-axis indicated the frequency of DILI prediction models with a specific performance metrics value.

To estimate model performance, 5-fold cross-validations were conducted on both 2-class and 3-class datasets. The results of 1000 iterations of 5-fold cross-validations on the 2-class dataset were summarized by the distributions of five performance metrics given in Fig. 2A. The average prediction accuracy, sensitivity, specificity, Matthews correlation coefficient (MCC), and balance accuracy were 72.9%, 62.8%, 79.8%, 0.432, and 71.3%, respectively, indicating the DF prediction models performed well compared to the reported DILI prediction models. The standard deviations of the five metrics among the 1000 cross-validations were very small (1.6%, 3.0%, 1.9%, 0.035, and 1.7% for accuracy, sensitivity, specificity, MCC, and balance accuracy, respectively), indicating the DF prediction models were stable with different training drugs and could be robust when applying to prediction of new compounds.

**Figure 3:**
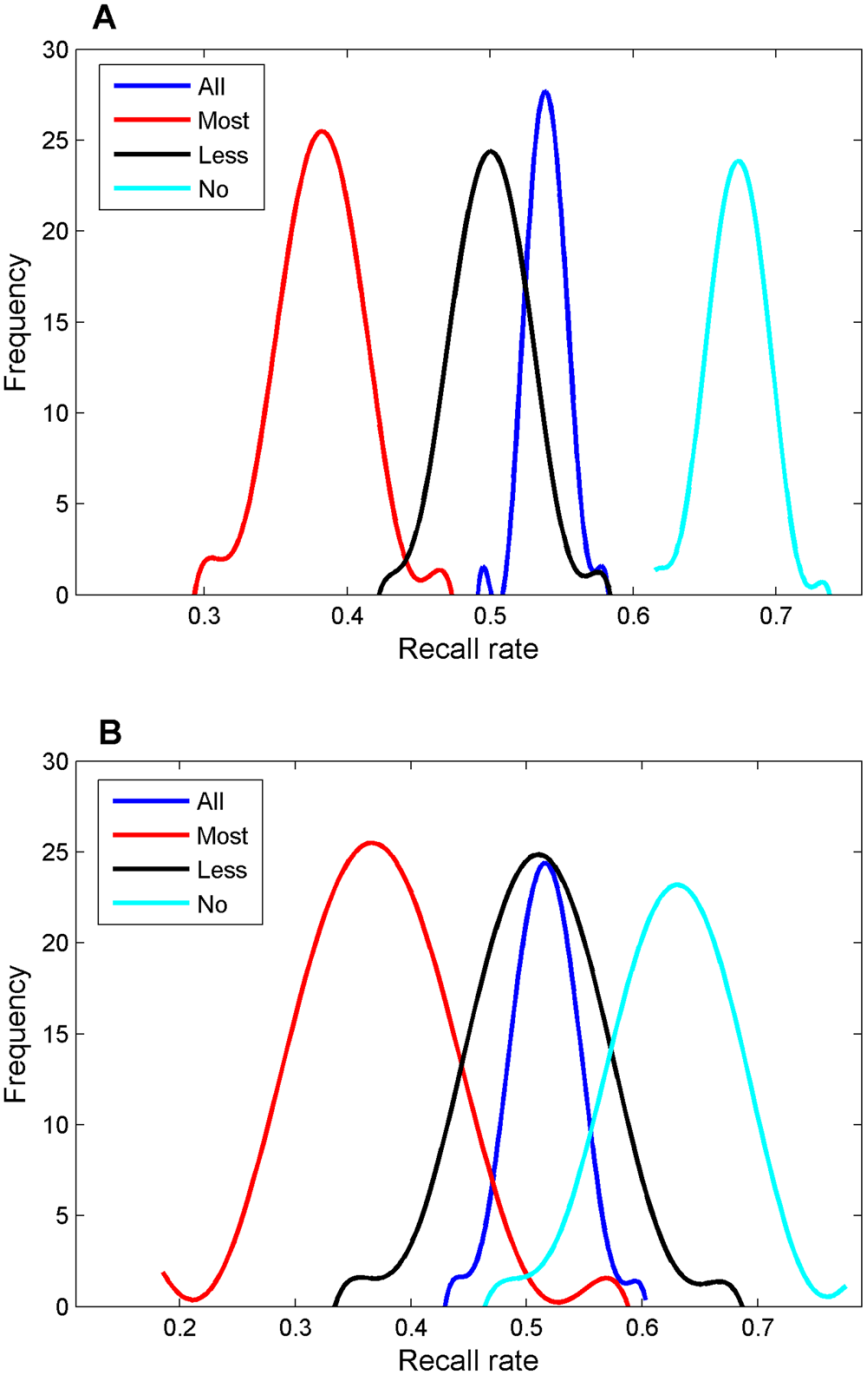
DILI risk prediction performance of the 5-fold cross-validations (**A**) and strategy A bootstrapping (**B**) on the 3-class dataset. Overall recall rates were plotted in blue curve, most-DILI in red, less-DILI in black, and no-DILI in cyan. The y-axis represented the frequency of DILI prediction models with a specific recall rate indicated at the x-axis.

The performances of the 1000 iterations of 5-fold cross-validations on the 3-class dataset were summarized using the distributions of different recall rates shown in Fig. 3A. The random recall rate for each of the three classes of samples is 33.3% if the prevalence of samples is balanced (37.2%, 25.4%, and 37.4% for no-, most-, and less-DILI, respectively, for the dataset in this study), the baseline similar with random accuracy of 50% for two classes of samples. The average recall rates for most-DILI, less-DILI, no-DILI, and all drugs were 37.9%, 52.7%, 64.6%, and 53.4%, respectively. It seemed that the 3-class DF prediction models had moderate prediction performance. However, lower recall rates were expected for a classification model with more than 2 class labels. Therefore, we examined the detailed predictions for the three classes of drugs and the results were given in Table 1. For the most-DILI drugs, 37.9% were correctly predicted as most-DILI drugs, which is slightly higher than the random recall rate (25.4%). For the remaining 62.1% wrongly predictions for the most-DILI drugs, 37.8% were predicted as less-DILI drugs and could be considered as reasonable predictions. Only 23.4% most-DILI drugs were incorrectly predicted as no-DILI drugs, indicating a low error rate for the 3-class DF models. For the less-DILI drugs, in addition to the 52.7% recall rate, another 20.8% were predicted as most-DILI drugs, and that could be considered as reasonable predictions because only 25.9% were predicted as no-DILI drugs. For the no-DILI drugs, 64.6% were correctly predicted as no-DILI drugs and 24.6% were predicted as less-DILI drugs. A very small portion, 10.3%, was predicted as most-DILI and it should be treated as the incorrect predictions. Of noted, 0.8% most-DILI, 0.6% less-DILI, and 0.5% no-DILI drugs could not be predicted for any of the three classes and were output as an unknown class by the DF models.

**Table 1:** Distribution of 3-class DILI predictions in cross validations and bootstrapping.

	Actual	Probability of Prediction			
		No-DILI	Most-DILI	Less-DILI	Unknown
Cross validations	No-DILI	0.6455	0.1029	0.2462	0.0054
	Most-DILI	0.2344	0.3793	0.3784	0.0079
	Less-DILI	0.2585	0.2083	0.5268	0.0064
Bootstrapping Strategy A	No-DILI	0.6259	0.1136	0.2525	0.0081
	Most-DILI	0.2385	0.3660	0.3844	0.0111
	Less-DILI	0.2699	0.2120	0.5080	0.0101
Bootstrapping Strategy B	No-DILI	0.6137	0.1176	0.2640	0.0046
	Most-DILI	0.2492	0.3532	0.3911	0.0065
	Less-DILI	0.2815	0.2117	0.5020	0.0048

### Bootstrapping

A different internal validation strategy, bootstrapping, was also applied to further estimate model performance. The performance of 1000 iterations of bootstrapping strategy A for the 2-class dataset was given in Fig. 2B. The average prediction accuracy, sensitivity, specificity, Matthews correlation coefficient (MCC), and balance accuracy were 70.9%, 60.3%, 78.4%, 0.393, and 69.3%, respectively, close to those of cross validations. Similar results were obtained using bootstrapping strategy B (Supplementary Figure S1). The bootstrapping results further revealed that the DF prediction models performed well compared to the reported DILI prediction models. The standard deviations of the five metrics among the 1000 bootstrapping validations were also small (3.2%, 6.6%, 4.4%, 0.067, and 3.4% in strategy A for accuracy, sensitivity, specificity, MCC, and balance accuracy, respectively), further indicating robustness of the DF prediction models.

The results of the 1000 iterations of bootstrapping strategy A validations on the 3-class dataset were plotted in the distributions of different recall rates shown in Fig. 3B. The average recall rates for most-DILI, less-DILI, no-DILI, and all drugs were 36.8%, 51.0%, 62.3%, and 51.6%, respectively. The results were similar with the five-fold cross-validations. Similar results were obtained using bootstrapping strategy B (Supplementary Figure S2). We also examined the detailed predictions for the three classes of drugs and the results were given in Table 1. Similar observations to the cross-cross validations were obtained. For the most-DILI drugs, 36.6% and 38.4% (strategy A) and 35.3% and 39.1% (strategy B) were predicted as most-DILI and less-DILI drugs, respectively. Only 23.9% (strategy A) and 24.9% (strategy B) most-DILI drugs were incorrectly predicted as no-DILI drugs. For the less-DILI drugs, 50.8% and 21.2% (strategy A) and 50.2% and 21.2% (strategy B) were predicted as less-DILI and most-DILI drugs, respectively. Only 27.0% (strategy A) and 28.2% (strategy B) were predicted as no-DILI drugs. For the no-DILI drugs, 62.6% (strategy A) and 61.4% (strategy B) were correctly predicted as no-DILI drugs and 25.3% (strategy A) and 26.4% (strategy B) were predicted as less-DILI drugs. A very small portion (11.4% for strategy A and 11.8% for strategy B) was predicted as most-DILI. Similar with the cross-validations, 1.1% and 0.7% most-DILI, 1.0% and 0.5% less-DILI, and 0.8% and 0.5% no-DILI drugs for bootstrapping strategy A and B could not be predicted as the training classes and were output as an unknown class by the DF models.

### Prediction confidence analysis

**Figure 4:**
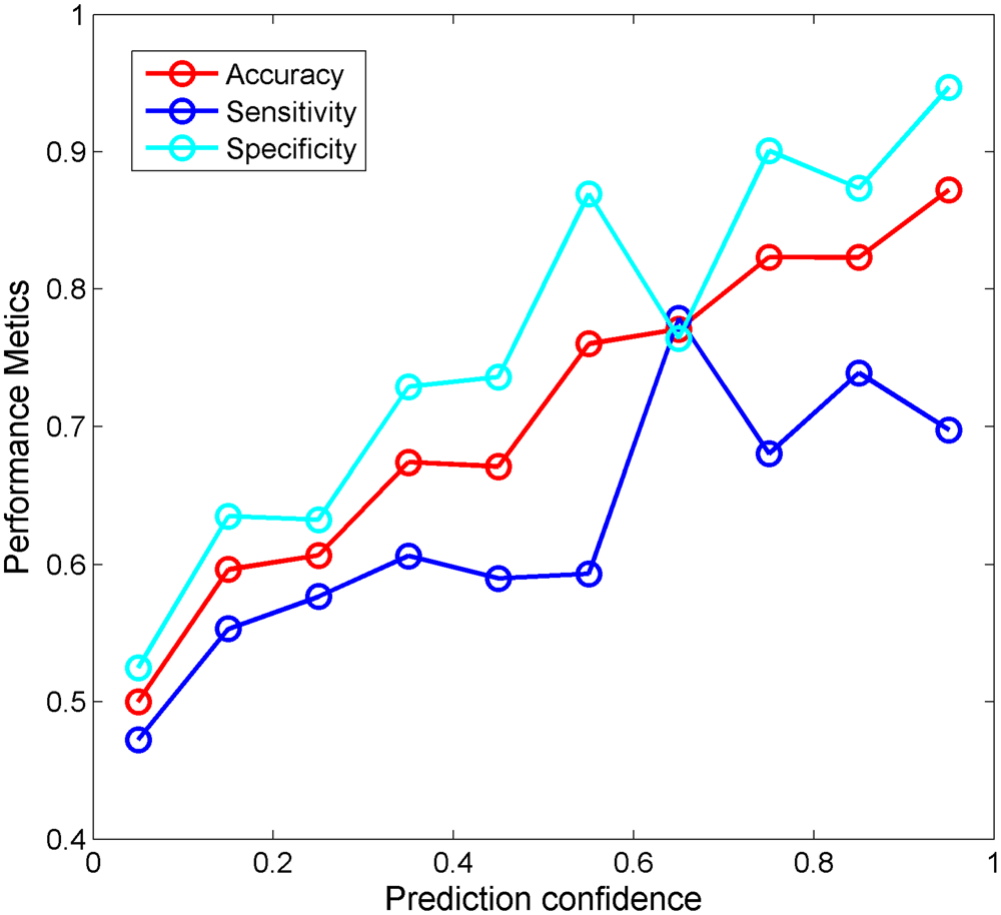
Prediction confidence analysis of the 5-fold cross-validations on the 2-class dataset. The prediction performance metrics were indicated by the y-axis. Prediction confidence was given at the x-axis. Prediction accuracy was plotted in red, sensitivity in blue, and specificity in cyan.

DF not only classifies a sample but also outputs a likelihood measure that quantitatively assesses confidence of the class prediction, which is how likely the sample should be in the assigned class. The confidence values of the predictions from the 1,000 iterations of 5-fold cross-validations on the 2-class dataset were calculated first. The predictions were then placed into 10 groups with 10 even prediction confidence bins between 0 and 1 based on their confidence values. The performance of DF models was assessed for each of the 10 groups of predictions. Accuracy, sensitivity, and specificity at different prediction confidence levels were calculated and plotted in Fig. 4. As the confidence (x-axis) increases, the prediction accuracy, sensitivity, and specificity (y-axis) increase. The confidence analysis results revealed that the higher the prediction confidence level is, the better the performance of predictions is.

**Figure 5:**
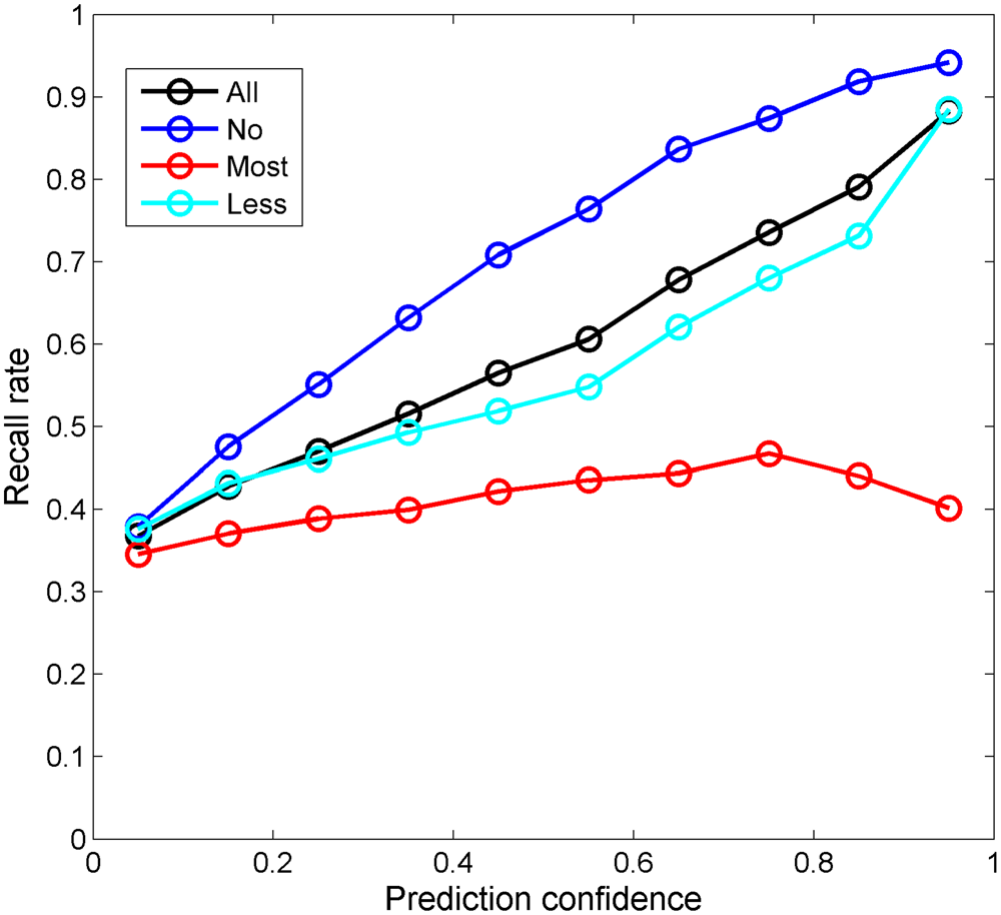
Prediction confidence analysis of the 5-fold cross-validations on the 3-class dataset. The prediction performance metrics were indicated by the y-axis. Prediction confidence was given at the x-axis. Overall recall rates were plotted in black, no-DILI recall rates in blue, most-DILI recall rates in red, less-DILI recall rates in cyan.

The confidence values of the predictions from the 1,000 iterations of 5-fold cross-validations on the 3-class dataset were also calculated. In a similar way, the 3-class predictions were placed into 10 groups with 10 even prediction confidence bins between 0 and 1 based on their confidence values. The performance of 3-class predictions was assessed for each of the 10 groups of predictions. The recall rates at different prediction confidence levels were calculated for no-DILI, most-DILI, less-DILI, and all drugs and plotted in Fig. 5. As the confidence (x-axis) increases, the recall rates (y-axis) for no-DILI and less-DILI drugs increase. However, the recall rate for most-DILI drugs is not improved. The confidence analysis results revealed that the higher the prediction confidence level is, the better the overall prediction performance for the 3-class DILI prediction models.

### Identification of chemical features associated with human DILI risk

The most frequently used chemical descriptors by the models are of informative to the human DILI risk. The chemical features represented by the informative molecular descriptors are important for the interpretation of human DILI risk of drugs. To identify the informative descriptors in the DF models during the 5-fold cross-validations, we first extracted the Mold2 descriptors that were used in the models. Then, the frequency for each of the Mold2 descriptors used by the DF models was calculated. The results for the 2-class models were plotted in Supplementary Figure S3. The top 10 most frequently used Mold2 descriptors were listed in Table 2. These top 10 Mold2 descriptors were deemed as the informative molecular descriptors to the 2-class prediction models. They characterize molecular size, shape, and physical-chemical properties of chemicals, implying that these properties of a drug are associated with the human DILI risk.

**Table 2:** Descriptors frequently used in the 2-class DILI prediction models.

Mold2 ID	Descriptor Description	Models
D144	Mean atomic van der Waals volume (scaled on Carbon atom)	6716
D504	Moran autocorrelation - lag 2/weighted by atomic polarizabilities	6442
D361	Ratio of multiple path counts to path counts	6337
D152	Mean atomic polarizability (scaled on Carbon-SP3 atom)	6330
D450	Geary autocorrelation - lag 4/weighted by atomic masses	6308
D448	Geary autocorrelation - lag 2/weighted by atomic masses	6215
D449	Geary autocorrelation - lag 3/weighted by atomic masses	6087
D123	Average weight of molecular	6054
D465	Geary autocorrelation - lag 3/weighted by atomic Sanderson electronegativities	6025
D464	Geary autocorrelation - lag 2/weighted by atomic Sanderson electronegativities	6006

In a similar way, the frequency of each Mold2 descriptor used in the 5-fold cross-validations for the 3-class dataset was calculated and is provided in Supplementary Figure S4. The top 10 most frequently used Mold2 descriptors were listed in Table 3 and were deemed as the informative molecular descriptors for the 3-class prediction models. These top 10 Mold2 descriptors are used to describe molecular size, shape, and physical-chemical properties of chemicals. Not surprising, comparing the informative descriptors between 2-class and 3-class prediction models revealed that seven of the top 10 are common. Therefore, characterizations of the informative descriptors imply that molecular size, shape, and physical-chemical properties of a drug contribute to human DILI risk.

**Table 3:** Descriptors frequently used in the 3-class DILI prediction models.

Mold2 ID	Descriptor Description	Models
D465	Geary autocorrelation - lag 3/weighted by atomic Sanderson electronegativities	20483
D448	Geary autocorrelation - lag 2/weighted by atomic masses	20396
D504	Moran autocorrelation - lag 2/weighted by atomic polarizabilities	20341
D152	Mean atomic polarizability (scaled on Carbon-SP3 atom)	20245
D457	Geary autocorrelation - lag 3/weighted by atomic van der Waals volumes	20172
D123	Average weight of molecular	20159
D450	Geary autocorrelation - lag 4/weighted by atomic masses	19988
D452	Geary autocorrelation - lag 6/weighted by atomic masses	19987
D144	Mean atomic van der Waals volume (scaled on Carbon atom)	19962
D503	Moran autocorrelation - lag 1/weighted by atomic polarizabilities	19939

### Permutation tests

For both 2-class and 3-class datasets, 1000 runs of permutation tests were conducted as illustrated in Fig. 1. The performance metrics (overall prediction accuracy, sensitivity, specificity, MCC, and balanced accuracy) of the 2-class prediction models were compared between the 5-fold cross-validations and the permutation tests and between the bootstrapping strategy A and permutations as shown in Supplementary Figures S5 and S6, respectively. The average prediction accuracy, sensitivity, specificity, MCC, and balanced accuracy of the 1000 permutation tests were 52.1%, 35.5%, 63.4%, −0.011, and 49.5%, respectively. The 1000 iterations of 5-fold cross-validation and bootstrapping strategy A had much larger related performance metrics than the permutation tests, indicating the 2-class human DILI risk prediction models were not generated by chance and had a very good prediction power (the performance metrics distributions are separated well between cross-validations and permutation tests and between and the bootstrapping strategy A and permutations as shown in Supplementary Figures S5 and S6).

The performances of the 1000 iterations of permutation tests on the 3-class dataset were summarized using the distributions of different recall rates and compared with the related distributions in the cross-validations and bootstrapping strategy A (Supplementary Figures S7 and S8). The average recall rates for most-DILI, less-DILI, no-DILI, and all drugs were 17.0%, 39.8%, 35.8%, and 32.5%, respectively. Comparing with the related performance metrics in the cross-validations (37.9%, 52.7%, 64.6%, and 53.4% of most-DILI, less-DILI, no-DILI, and all drugs were correctly recalled) and bootstrapping strategy A (36.8%, 51.0%, 62.7%, and 51.6% of most-DILI, less-DILI, no-DILI, and all drugs were correctly recalled) demonstrated that the 3-class human DILI risk prediction models were not obtained by chance and did show a good predictive power (the performance metrics distributions are separated well between cross-validations and permutation tests and between bootstrapping strategy A and permutations as shown in Supplementary Figures S7 and S8).

### Application cases

**Figure 6:**
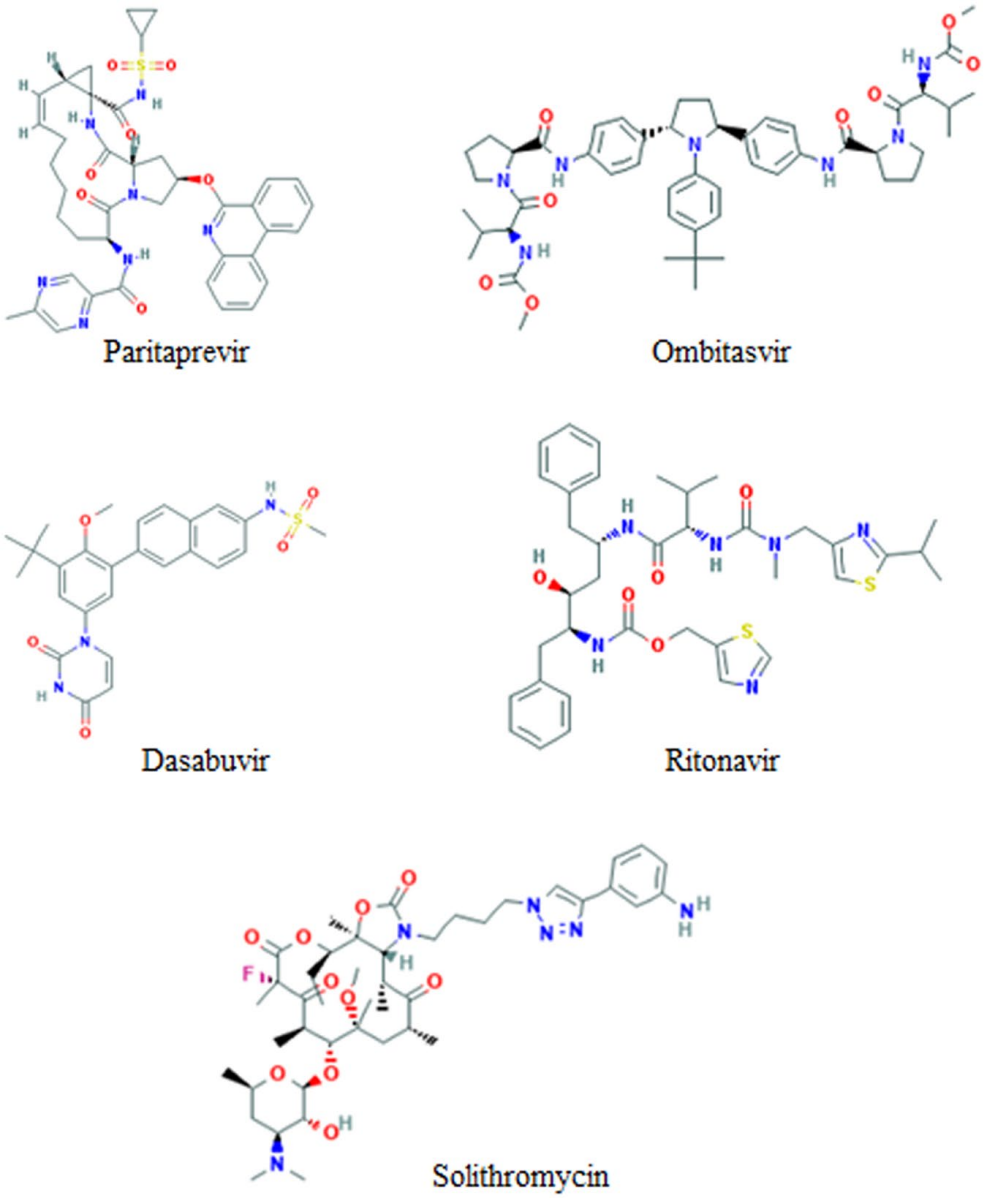
Chemical structures of the five drug ingredients. The structures were downloaded from PubChem. The names of the five drug ingredients were given under the related structures.

We tested the models on three DILI cases reviewed by the FDA in recent years. The first two cases are about hepatitis C treatment drugs, Viekira Pak and Technivie. The FDA recently gave warnings on both drugs for their cause of serious liver injury in patients. Their DILI cases are reported to the FDA Adverse Event Reporting System (FAERS) database (http://www.fda.gov/Safety/MedWatch/SafetyInformation/SafetyAlertsforHuman MedicalProducts/ucm468757.htm, accessed on Jan 3, 2017). Viekira Pak was approved by FDA in December 2014 and has four ingredients: paritaprevir, ombitasvir, dasabuvir, and ritonavir. The structures of the four chemicals were given in Fig. 6. Technivie was approved in July 2015 and has three ingredients (i.e., paritaprevir, ombitasvir, and ritonavir) that are also in Viekira Pak. The third case is about the experimental drug for community-acquired pneumonia treatment, solithromycin. A preliminary review by FDA revealed a significant safety signal for hepatotoxicity of the drug (http://www.reuters.com/article/us-cempra-antibiotic-fda-idUSKBN12×1HW, accessed on Jan 3, 2017). The molecular structure of solithromycin was provided in Fig. 6. The five chemicals were predicted using both 2-class and 3-class DILI prediction models. The predictions and related confidence values from the two models were listed in Table 4. The 2-class model predicted ombitasvir with no-DILI risk and the other four to potentially cause DILI in humans. The 3-class model predicted paritaprevir to less-DILI risk in humans and the rest four ingredients to have most-DILI risk in humans.

**Table 4:** Prediction results.

Model		Paritaprevir	Ombitasvir	Dasabuvir	Ritonavir	Solithromycin
Type	Result					
2-Class	Prediction	most-DILI	no-DILI	most-DILI	most-DILI	most-DILI
	Confidence	0.232	0.272	0.994	0.504	0.098
3-Class	Prediction	less-DILI	most-DILI	most-DILI	most-DILI	most-DILI
	Confidence	0.332	0.002	0.833	0.74	0.32

## Discussion

Human DILI risk remains a challenge for the industry and regulatory agencies because DILI is a complicate safety concern owing to diverse mechanisms, various severity levels, variation in population groups, and difficulty in the classification of DILI risk, especially for the drugs that have been approved for marketing for a short time. Predicting potential of human DILI risk is needed for decreasing the likelihood of introduction of DILI drugs into the market and reducing development cost for the industry to early terminate the development of drug candidates that are in high probability to cause DILI in clinical trials. Though various experimental methods such as *in vitro* and *in vivo* assays have been developed and used in current practices, *in silico* models have been gained attraction to the scientists in the field as development and validation of *in silico* models usually take less time and are at lower cost than the experimental methods. Many *in silico* DILI prediction models have been reported^3–9^; however, the reported DILI prediction models have various limitations for employment in regulatory setting and inclusion in drug development^19^. Therefore, efforts are needed to develop new DILI prediction models for improving prediction performance.

Many factors impact performance of *in silico* DILI prediction models. One of the most important factors contributing to the success of a DILI prediction model is reliability of DILI risk classification for the drugs that are used to train prediction models. Multiple methods for classification of drugs into DILI or no-DILI have been proposed and applied to the development of *in silico* DILI prediction models^17,18^. We previously suggested a new classification method to classify drugs into three classes of human DILI risk (most-DILI, less-DILI, and no-DILI) based on the FDA-approved drug labeling documents^27^. We then developed QSAR models for prediction of human DILI risk using the most-DILI and no-DILI drugs and the models showed a slight improvement compared to other models^3^. Severity level of DILI varies among drugs and is a very important metric for measuring human DILI risk. Therefore, we improved our human DILI risk classification for a large set of FDA-approved drugs by integration of severity levels and FDA-approved drug labels, yielding the DILIrank dataset^24^. The 2-class DILI prediction models developed using DILIrank had a better performance (average accuracy 72.9% and 70.9% for cross-validations and bootstrapping strategy A) compared to our previous models (average accuracy 69.7% for cross-validations) that were constructed using the drugs classified by the FDA-approved drug labels only^3^, indicating that inclusion of DILI severity level information in human DILI risk classification did improve reliability of DILI prediction models.

The *in silico* models reported in the literature were mainly focused on binary prediction, that is predicting a drug to have DILI risk or not. It is more challenging to develop a model to predict multiple levels of DILI risk, a multi-class prediction model to predict a drug into one of more than two classes. First, classification of drugs into multiple DILI risk levels is more difficult than assign drugs as DILI or not DILI drugs. Of note, mathematically training a multi-class prediction model is more arduous than constructing a 2-class prediction model. DILIrank dataset provided three classes of human DILI risk for a big number of drugs^24^. In addition, we previously developed a machine learning approach for the development of multi-class prediction models^26^, enabling us to develop the 3-class human DILI risk prediction models. The distributions of the 3-class predictions from the cross-validations and bootstrapping (Table 1) revealed that, for the most-DILI risk drugs, only 23.4% and 23.9% of them were incorrectly predicted as no-DILI drugs by the 3-class models, better than the performance of the 2-class DILI prediction models. The same observation was found for no-DILI drugs. The 3-class models predicted only 10.3% (cross-validation) and 11.4% (bootstrapping) of the drugs as most-DILI drugs, while the 2-class models had 20.2% of the drugs predicted as most-DILI drugs. Hence, our results demonstrated the first 3-class DILI risk prediction models developed to our knowledge, not only had a higher resolution in prediction of human DILI (most-DILI, less-DILI, and no-DILI) than the 2-class models (most-DILI and no-DILI) concern but also had a better ability to differentiate most-DILI drugs from no-DILI drugs.

Different from other classification algorithm (such as for most binary classification) where a sample belongs one of the classes, the DF methodology allows a sample that could belong none of the classes which reflects the real biological phenomena. Examination of the 3-class predictions from the cross-validations and bootstrapping (Table 1) indicated that some drugs (very few) were predicted to be not in any of the three classes and were classified as “unknown”. This unique feature of our multi-class DF algorithm already showed its utilization in the clinical study to discover new diseases or new subtypes of a disease^26^. The results of this study demonstrated the potential of application of this unique feature in drug safety evaluation. The unknown class prediction could be used as a warning for further confirmation of the real class of the predicted drug, having the potential for discovery of new safety concern such as cardiovascular toxic drugs that were wrongly classified as one of the three DILI classes.

Performance of an *in silico* prediction model is dependent on many factors such as size of the training set, quality of dependent variable (DILI classification in current study), characteristics of structural representations, and modeling algorithms. All the reported *in silico* DILI prediction models had some limitations to be proactively applied for new drugs. Therefore, to facilitate the regulatory decisions, it is important for a model to provide a confidence measure as an estimation of the vitality of prediction^28^. A DF model not only provides predictions but also calculates a metric. This information provides the likelihood measure for confidence levels of the predictions. Prediction confidence analysis showed that the 2-class models predicted human DILI risk accurately for some drugs but did not perform well for some other drugs. The higher the prediction confidence level ensures the accuracy of DILI risk prediction and is shown in Fig. 4. Similar trends were observed for the 3-class DILI prediction models except for most-DILI related predictions (Fig. 5). As discussed above, distinguishing most-DILI from less-DILI is challenging. The 3-class models would have similar confidence distributions of the 2-class models if the predictions of most-DILI as less-DILI were considered as correct. The DILI prediction confidence analysis suggested that human DILI risk predictions should be considered together with their prediction confidence levels in practices.

Performance metrics are used to measure the goodness of a predictive model. However, performance metrics values are also dependent not only on the model but also on the composition of samples used to train and test the model. One practice for better assessment of predictive power of a model is to conduct permutation tests using the same dataset. The performance metrics for the 3-class human DILI prediction models (Fig. 3) seemed not as good as the ones for the 2-class models (Fig. 2). Comparison with the permutation tests (Supplementary Figures S5, S6, S7 and S8) revealed that both the 2-class and 3-class DILI prediction models have good predictive powers. The differences in performance metrics between the cross-validations (or bootstrapping) and the permutation tests are statistically significant, with p-values < 0.0001 for both the 2-class and 3-class models, indicating that the models have predictive power and the modeling process is coded correctly. Our results support that permutation tests should be included in practices for development of *in silico* prediction models.

DF algorithm integrates variable selection into model development. The frequency of a chemical descriptor used for models in the cross-validations indicated importance of the descriptor for predicting human DILI risk of drugs. The top frequently used Mold2 descriptors are the descriptions of chemical features related to molecular size, shape, and physical-chemical properties, for both the 2-class and 3-class models. Molecular size and shape were known as the critical determinants for chemical transport^29^. They are crucial for metabolism of drugs in the body, which is directly related to DILI and supportive of our previous findings^4^. The identified physical-chemical properties such as electronegativity and polarizability are related to the off-target binding capability of chemicals which are necessary for assessing DILI potential of drugs^30,31^.

Validation of predictive models is an essential component for applications of the models. Different validation methods could be used for this purpose. Bootstrapping has been used as a method for assessing performance of predictive models^32,33^. Bootstrapping and k-fold cross-validations are considered as internal validations because the training and testing datasets are obtained from the same original dataset. They are widely used in the development of DILI prediction models^3,5,7,17,18,34^. The estimation of performance using a small number of bootstrapping or k-fold cross-validations is dependent at the bootstrapping or division of a dataset into the training and testing sets^26^. A big number of bootstrapping and cross-validations are needed to reach a statistically robust estimation of model performance. We conducted 1000 iterations of bootstrapping and 5-fold cross-validations to estimate the performance of the 2-class and 3-class DILI risk prediction models. With such a big number of validations, the chance correlations that could be introduced in the bootstrapping and division of a dataset into training and testing sets were reduced as indicated by the small standard deviations yielded in the 1000 results. External validations (validation using external datasets) are considered as more stringent validations and should be more similar with real applicability of the models. Recently, FDA issued warnings on DILI risk for two drugs on the market and one experimental drug. The FDA liver toxicity working group members suggested us to test those drugs using the DILI prediction models developed in this study. The predictions of the five ingredients of those drugs given in Table 4 suggested that the human DILI risk prediction models would help supporting regulatory decision making on those drugs. Those three drugs were either on the market for very short time approved by the FDA in December 2014 (Viekira Pak) and July 2015 (Technivie) or not even on the market (experimental drug). All drugs were predicted for DILI at a reasonable confidence level. Hence, the models could be expected to facilitate regulatory science in drug safety review and to help pharmaceutical industry in decision of termination of development of drug candidates.

## Materials and Methods

### Datasets

DILIrank dataset contains 183 most-DILI, 270 less-DILI, and 268 no-DILI risk drugs^24^. All 721 drugs were used for 3-class DILI prediction model development, termed as 3-class dataset. The 2-class dataset was formed by the 183 most-DILI drugs and 268 no-DILI drugs. The names, CAS numbers, SMILES codes, and DILI risk classifications for the 721 drugs were listed in Supplementary Table S1. The two-dimensional (2D) chemical structures of the 721 drugs were generated from the SMILES codes using the online SMILES translator and structure file generator (http://cactus.nci.nih.gov/translate/, accessed on June 14, 2016) developed by the National Cancer Institute (NCI) at the National Institutes of Health (NIH). A structure description file (SDF) that contains the structures of the 721 drugs was obtained from NCI and used for molecular descriptors calculation.

For the five ingredients of three drugs used to test applicability of our DILI prediction models, SMILES codes were obtained by searching the chemicals in PubChem database (https://pubchem.ncbi.nlm.nih.gov/, accessed on December 18, 2016). The same NCI structure generator was used to construct 2D structures from the SMILES codes.

### Molecular descriptors

Molecular descriptors of the 721 drugs from DILIrink and the five drug ingredients were generated using Mold2 (http://www.fda.gov/ScienceResearch/BioinformaticsTools/Mold2/default.htm). Mold2 is a free software package that calculates molecular descriptors from 2D chemical structures^35^. It adopts some fast algorithms from structure elucidation system ESSESA^36^. This software has been demonstrated to be a useful tool for QSAR model development^3,37–41^. In brief, 777 Mold2 descriptors were first calculated for each of the 721 drug molecules and the 5 drug ingredients. Then, the Mold2 descriptors were preprocessed to remove those with constant values across all the 721 drugs.

### DF model

**Figure 7:**
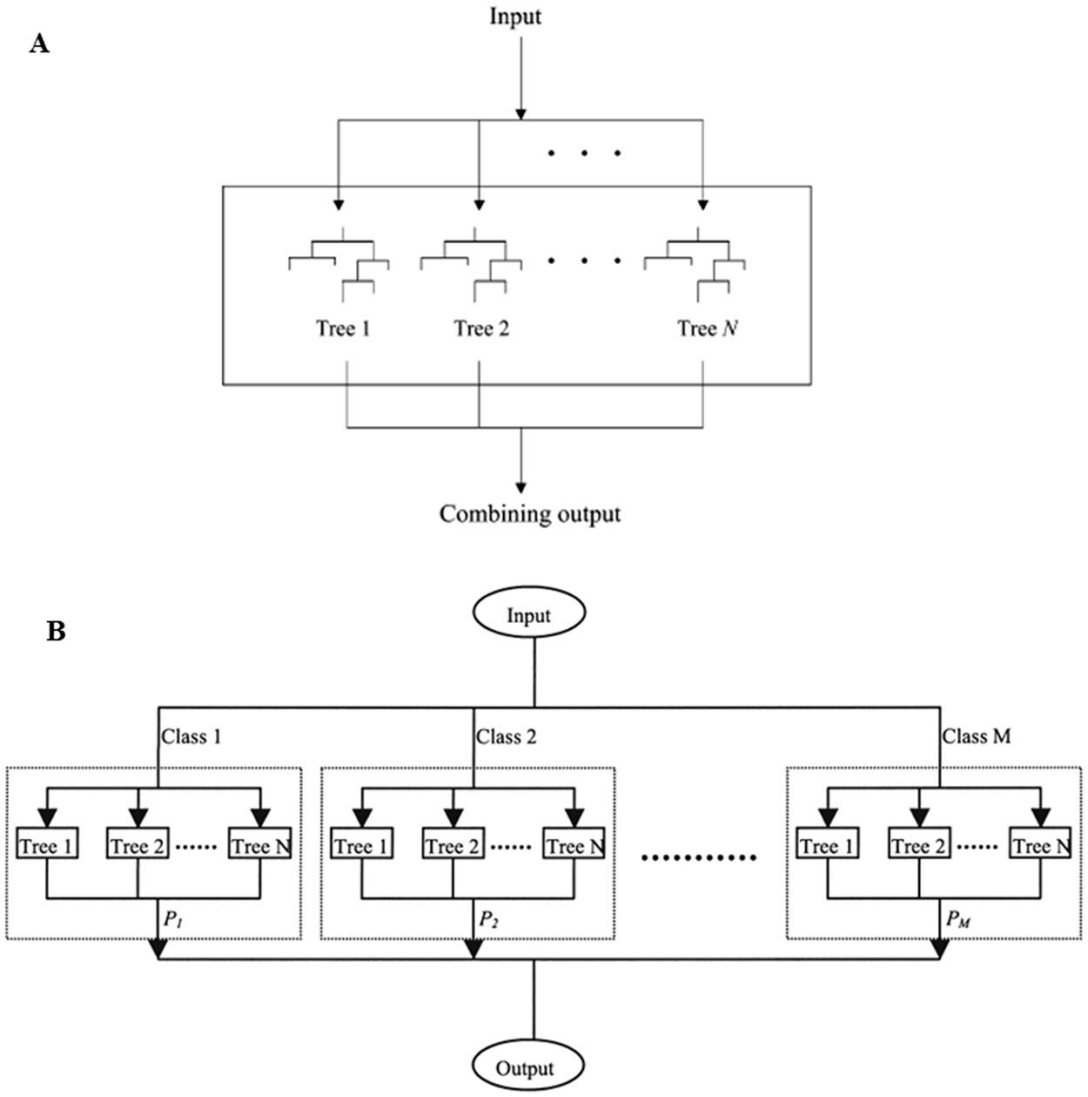
DF algorithmic flowcharts. The flowchart for 2-class DF was illustrated in (**A**). Accurate decision tree models were constructed from an input dataset using diverse independent variables (Mold2 descriptors in this study). Predicting a drug is to combine predictions of the drug from the N decision tree models by averaging the N probabilities of the decision tree models to obtain a consensus probability for the drug to be most-DILI. The flowchart for multi-class DF was illustrated in (**B**). First, for each class, a 2-class (all other classes are combined as another class) DF model is generated as illustrated in (**A**). To predict a drug is to compare the probabilities of the drug from multiple DF models. The drug is predicted as the class with the largest probability value. Unknown is assigned to the drug if more than one values are equally the largest.

QSAR models can be developed using a variety of methods such as pharmacophore modeling^42^, molecular docking^43–46^ and machine learning^47^. In this study, the DILI risk prediction models were built using the pattern recognition algorithm DF. A 2-class version is available online (http://www.fda.gov/ScienceResearch/BioinformaticsTools/DecisionForest/default.htm). DF employs a consensus modeling of multiple decision tree models in a different way compared to the Random Forests algorithm^48^. Although both DF and Random Forests combine decision trees, the decision trees are constructed in different ways. First, DF construct a small number of deep decision trees using all samples and variables, while Random Forests build a big number of shallow decision trees using part of samples through bootstrapping and a small portion of variables by random selection. Second, the diversity of decision trees in DF are achieved by using different variables to ensure benefit of consensus model, while Random Forests ensures contributions of decision trees by randomization. The 2-class DILI prediction models were constructed using the DF algorithm that separately generates decision tree models with different descriptors to ensure heterogeneity of the member models^25,49^. Figure 7A explained the principles of DF algorithm. DF first generated a set of decision tree models using diverse sets of Mold2 descriptors. The probability values to predict a drug as DILI risk drug from the decision tree models were combined with equal weights to calculate a consensus probability value for measuring the likelihood of the drug to be a DILI risk one. The 3-class DILI prediction models were developed using the multi-class DF algorithm^26^. The principal of the multi-class DF algorithm was illustrated in Fig. 7B. In brief, a 2-class DF model was constructed for each of the three classes (most-DILI, less-DILI, and no-DILI) by combining the samples of the other two classes into one class. The probability value from the DF model was used to predict the likelihood of a drug in the class modeled. The final prediction for the drug depends on the three probability values from the three DF models using an approach of winner takes all. When winners are more than one, assigning to only one of the three classes was not possible and the 3-class DF model predicted the class of the drug to be unknown. Both the 2-class and 3-class DILI risk prediction models were constructed using the same algorithmic parameters: number of trees in a 2-class DF model was 5; the minimum number of samples to split a node was 10; decision trees can be pruned up to 2 levels; and Gini’s diversity index was used to split nodes.

### Cross-validations

To assess the performance of the DILI risk prediction models, 5-fold cross-validations were conducted as shown in Fig. 1. In one 5-fold cross-validation, the drugs (451 for 2-class and 721 for 3-class models) were randomly divided into five groups. Four of the five groups were used to build a DILI prediction model. The developed prediction model was then used to predict human DILI risk for the drugs in the remaining group. This process was iterated so that each of the five groups was used as the testing set once and only once. The DILI risk prediction results obtained from all five testing groups were then combined for calculating performance metrics (accuracy, sensitivity, specificity, MCC and balanced accuracy for 2-class prediction models or recall rates for 3-class prediction models). To reach a statistically robust estimate for the DILI prediction models, 5-fold cross-validation was repeated 1000 time by randomly dividing the whole dataset into 5 groups using different random seeds.

### Bootstrapping

Bootstrapping strategy was used to validate the performance of the models as shown in Fig. 1. In one bootstrapping, the original drugs (451 for 2-class and 721 for 3-class models) were randomly resampled with replacement of the samples that were strapped in the boot. Two bootstrapping strategies were adopted. Strategy A ensures a fixed portion (63.2% used in this study) of the original drugs were in the boot with various copies of the same drugs. More specifically, in strategy A, a drug and its Mold2 descriptors were randomly selected to add into the boot from the original dataset and marked as strapped. The original data remained the same (the result of replacement of the strapped sample). The unique drugs (not size of the boot) in the boot were counted to make decision on bootstrapping: repeat the process if less than the fixed portion (63.2%) and stop it otherwise. The drugs in the boot were then used to train a human DILI risk prediction model and the drugs out of the boot were then used to challenge the model. The prediction results on the drugs out of the boot were compared with their actual DILI classes to estimate the performance of the model. To reach a statistically robust estimate for the DILI prediction models, bootstrapping in strategy A was repeated 1000 time by randomly resampling using different sets of random seeds. The size distribution of the 1000 boots was shown in Supplementary Figure S9 for 2-class models and Supplementary Figure S10 for 3-class models. The average boot size is 452 for 2-class models and 722 for 3-class models. Strategy B is typical in bootstrapping. In contrast to strategy A in which boot sizes vary but the number of samples out of boot remains the same, strategy B fixes the size of boots but allows different numbers of samples out of the boots. More specifically, strategy B randomly selected a sample to put into a boot with replacement of the selected samples (thus the dataset remained the same). This process was repeated until the boot size reached the same of the original dataset. Bootstrapping in strategy B was repeated 2000 time by randomly resampling using different sets of random seeds. The size distribution of the 2000 testing sets (drugs out of the boots) was shown in Supplementary Figure S11 for 2-class models and Supplementary Figure S12 for 3-class models. The average size of the testing sets is 240 for 2-class models and 383 for 3-class models.

### Performance metrics

Predictions from each of the 1000 iterations of 5-fold cross-validations were compared with their actual DILI risk classifications to calculate performance metrics. For the 2-class DILI prediction models, each 5-fold cross-validation resulted in a 2 × 2 confusion matrix as shown in Table 5.

**Table 5:** Confusion matrix of predictions of 2-class models from a 5-fold cross validation.

	Actual most-DILI	Actual no-DILI
Predict most-DILI	TP (true positive)	FP (false positive)
Predict no-DILI	FN (false negative)	TN (true negative)

To assess performance of models in the cross-validation, prediction accuracy, sensitivity, specificity, MCC and balanced accuracy were calculated based on the 2 × 2 confusion matrix using equations (1) to (5).

1 $$Accuracy=\frac{TP+TN}{TP+TN+FP+FN}$$

2 $$Sensitivity=\frac{TP}{TP+FN}$$

3 $$Specificity=\frac{TN}{TN+FP}$$

4 $$MCC=\frac{TP\times TN-FP\times FN}{\sqrt{(TP+FP)(TP+FN)(TN+FP)(TN+FN)}}$$

5 $$Balanced\cdot Accuracy=\frac{TP(TN+FP)+TN(TP+FN)}{2(TP+FN)(TN+FP)}$$

For the 3-class DILI prediction models, each 5-fold cross-validation resulted in a 3 × 4 confusion matrix as shown in Table 6.

**Table 6:** Confusion matrix of predictions of 3-class models from a 5-fold cross validation.

	Actual most-DILI	Actual less-DILI	Actual no-DILI
Predict most-DILI	MM	LM	NM
Predict less-DILI	ML	LL	NL
Predict no-DILI	MN	LN	NN
Predict unknown	MU	LU	NU

The 2-letter symbols used in the confusion matrix indicate the types of predictions. The first letter represents the actual DILI classes (M, L, and N for most-DILI, less-DILI, and no-DILI) of drugs. The second letter denotes predictions from the models (M, L, N, and U for most-DILI, less-DILI, no-DILI, and unknown). Performance metrics (most-DILI recall rate RR^m^, less-DILI recall rate RR^l^, no-DILI recall rate RR^n^, and overall recall rate RR°) were calculated based on the confusion matrix using equations (6) to (9).

6 $$R{R}^{m}=\frac{MM}{MM+ML+MN+MU}$$

7 $$R{R}^{l}=\frac{LL}{LM+LL+LN+LU}$$

8 $$R{R}^{n}=\frac{NN}{NM+NL+NN+MU}$$

9 $$R{R}^{o}=\frac{MM+LL+NN}{MM+ML+MN+MU+LM+LL+LN+LU+NM+NL+NN+NU}$$

### Permutation tests

Permutation analysis is a practice that can be used to determine whether predictive performance estimated for a model using internal validations such as cross-validations is due to chance correlations in the dataset used. It can also help assess predictive power of a model in real applications. We conducted permutation tests for both 2-class and 3-class DILI classification datasets as illustrated in Fig. 1. In brief, in a permutation test, the human DILI risk classes (most-DILI and no-DILI for 2-class models; most-DILI, less-DILI and no-DILI for 3-class models) of drugs in the actual dataset were randomly shuffled while the chemical descriptors remained the same to generate a permutated dataset. Then, 5-fold cross-validation was performed on the permutated dataset. The permutation test was iterated 1,000 times by using different random seed to randomly shuffle the actual DILI risk classes to reach a statistically robust estimate for the performance that could be expected for a random model constructed purely from chance correlations.

### Prediction confidence analysis

In the 5-fold cross-validations, the prediction from a 2-class DILI prediction model for a drug was a probability value, p, that was used to estimate the likelihood of the drug as most-DILI (p ≥ 0.5) or no-DILI (p < 0.5) drug. A 3-class DILI prediction model output three probability values for a drug. They were used to estimate how likely the drug can be predicted as most-DILI, less-DILI, and no-DILI drug. Final prediction for the drug was the class having the maximum probability value, p. Therefore, p indicates confidence of a prediction. It is expected that predictions with higher prediction confidence should be more reliable than predictions with lower prediction confidence. We examined the relationship between prediction performance and prediction confidence using the results from 1,000 iterations of 5-fold cross-validations for both 2-class and 3-class datasets. For each of the predictions, a prediction confidence was calculated using equation (10).

10 $$Confidence=\frac{|p-0.5|}{0.5}$$

The calculated confidence was a value between 0 and 1. The larger the confidence value, the more reliable was the DILI risk prediction. In the confidence analyses for 2-class and 3-class models, predictions from the 5-fold cross-validations were placed into 10 groups based on their prediction confidence values. For each of the 10 groups of predictions, prediction performance metrics (accuracy, sensitivity and specificity for 2-class models; recall rates for all drugs and each of the three classes) were calculated by comparing the predictions with the actual DILI risk class data. At last, performance metrics of the DILI risk predictions were plotted against prediction confidence.

### Identification of informative descriptors

Generally, QSAR models are constructed for the purpose of prediction. However, interpretation of a QSAR model is also an important aspect for determination of goodness of the model. Mechanistic understanding of a QSAR model is dependent at the ability to interpret the molecular descriptors used in the QSAR model. To better understand the chemical features that play vital roles in DILI, the Mold2 descriptors used in the DILI risk prediction models were examined to identify informative Mold2 descriptors. First, a frequency was calculated for each Mold2 descriptor based on the models generated in the 1000 iterations of 5-fold cross-validations. All Mold2 descriptors were then ranked according to their frequency values. The most frequently used Mold2 descriptors were identified as the informative molecular descriptors and should be important in estimation of human DILI risk.

### Prediction cases

The *in silico* DILI prediction models reported in the literature were validated through different approaches such as hold-out, cross-validations, and external validations. All practiced validations were based on retroactive data. To demonstrate the applicability of the DILI risk prediction models developed in this study, we predicted DILI risk potential of some drug products on the market and in experiment that had warnings very recently issued by the FDA. Five ingredients in three drug products were predicted. First, SMILES codes of the five drug ingredients were downloaded from PubChem database. The 2D structures of the five chemicals were generated using the online SMILES translator and structure file generator from NCI. DILI prediction models were generated using the whole training datasets (2-class and 3-class datasets). Finally, the prediction models were used to predict human DILI risk potential of the five drug ingredients. For each drug ingredient, prediction confidence was also calculated for both 2-class and 3-class models prediction.

### Disclaimer

The findings and conclusions in this article have not been formally disseminated by the US Food and Drug Administration (F.D.A.) and should not be construed to represent the F.D.A. determination or policy.

## Electronic supplementary material

Supplementary Information 
