# Supplementary material for: Development of Decision Forest Models for Prediction of Drug-Induced Liver Injury in Humans Using A Large Set of FDA-approved Drugs
Source: Sci Rep. 2017 Dec 11;7:17311. doi: 10.1038/s41598-017-17701-7 (PMC5725422; doi:10.1038/s41598-017-17701-7)
Supplement: Supplementary file 1 — Supplementary Information [file 41598_2017_17701_MOESM1_ESM.doc]

**Supplementary Figures**

**Development of Decision Forest Models for Prediction of Drug-Induced Liver Injury in Humans Using A Large Set of FDA-approved Drugs**

Huixiao Hong*, Shraddha Thakkar, Minjun Chen, Weida Tong*

Division of Bioinformatics and Biostatistics, National Center for Toxicological Research, US Food and Drug Administration, Jefferson, Arkansas 72079, USA

^*^Correspondence should be addressed to Dr. Huixiao Hong ([huixiao.hong@fda.hhs.gov](mailto:huixiao.hong@fda.hhs.gov)) and Dr. Weida Tong (Weida.Tong@fda.hhs.gov): 3900 NCTR Road, Jefferson, AR 72079, USA


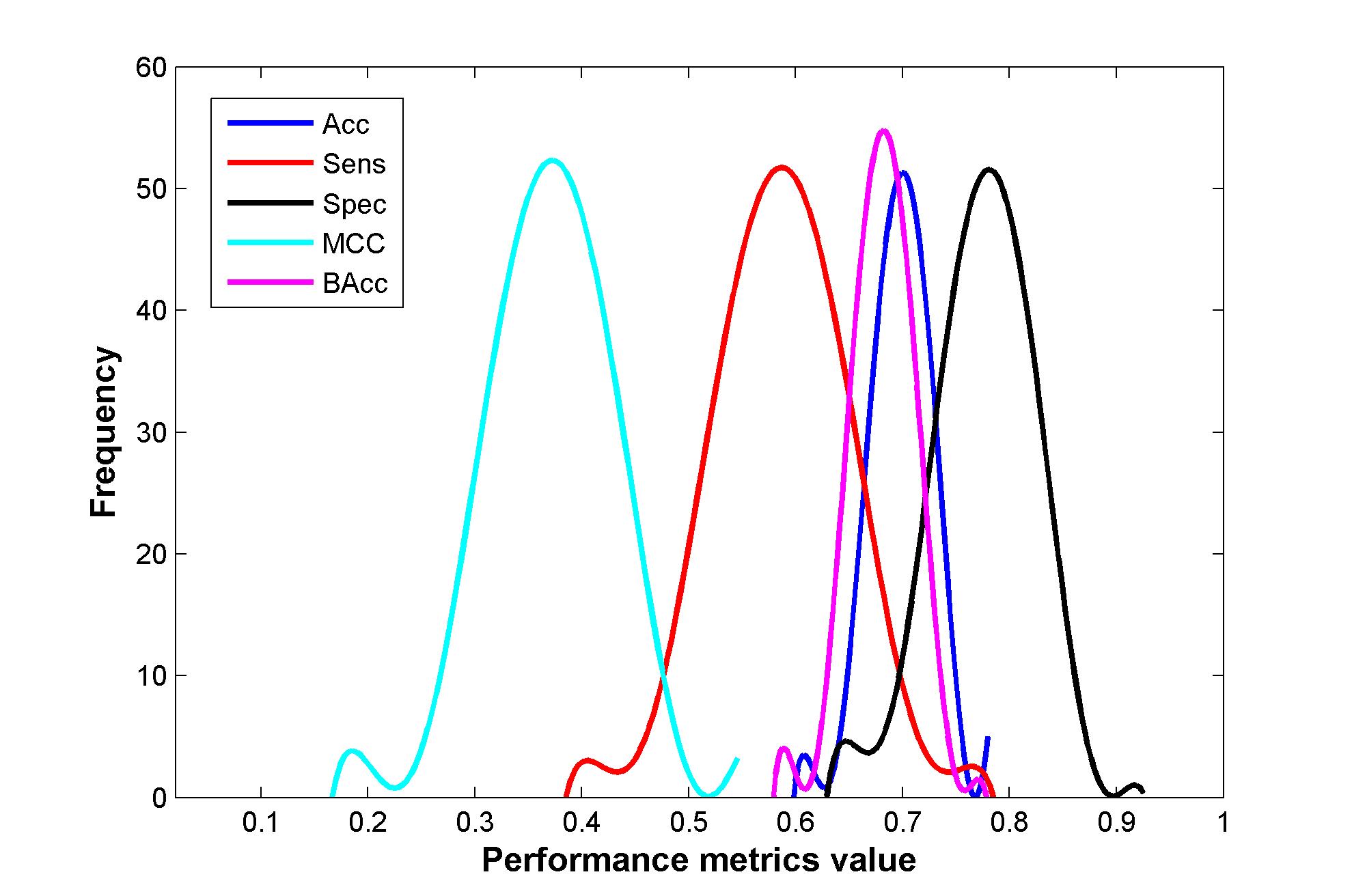


**Figure S1**. Bootstrapping (strategy B) on the 2-class dataset. Distributions of DILI prediction accuracy (blue, 0.698±0.028), sensitivity (red, 0.586±0.063), specificity (black, 0.777±0.046), MCC (cyan, 0.370±0.058) and balanced accuracy (magenta, 0.682±0.029) of the 2000 iterations of strategy B bootstrapping were plotted. Performance metrics values were given at the x-axis and the y-axis indicated the frequency of DILI prediction models with a specific performance metrics value.


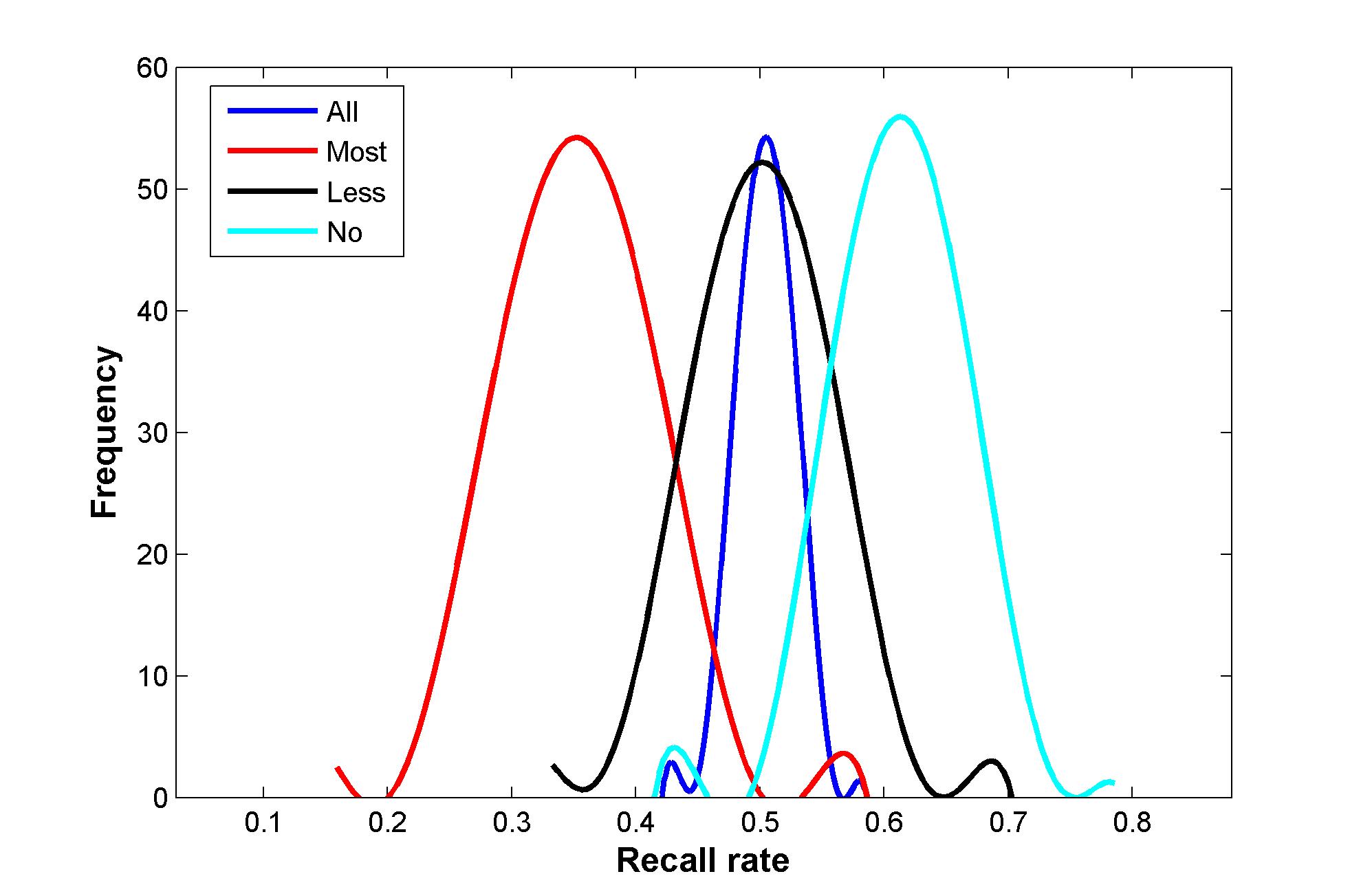


**Figure S2**. Bootstrapping (strategy B) on the 3-class dataset. Overall recall rates (0.502±0.023) were plotted in blue curve, most-DILI (0.353±0.061) in red, less-DILI (0.502±0.056) in black, and no-DILI (0.614±0.051) in cyan. The y-axis represented the frequency of DILI prediction models with a specific recall rate indicated at the x-axis.


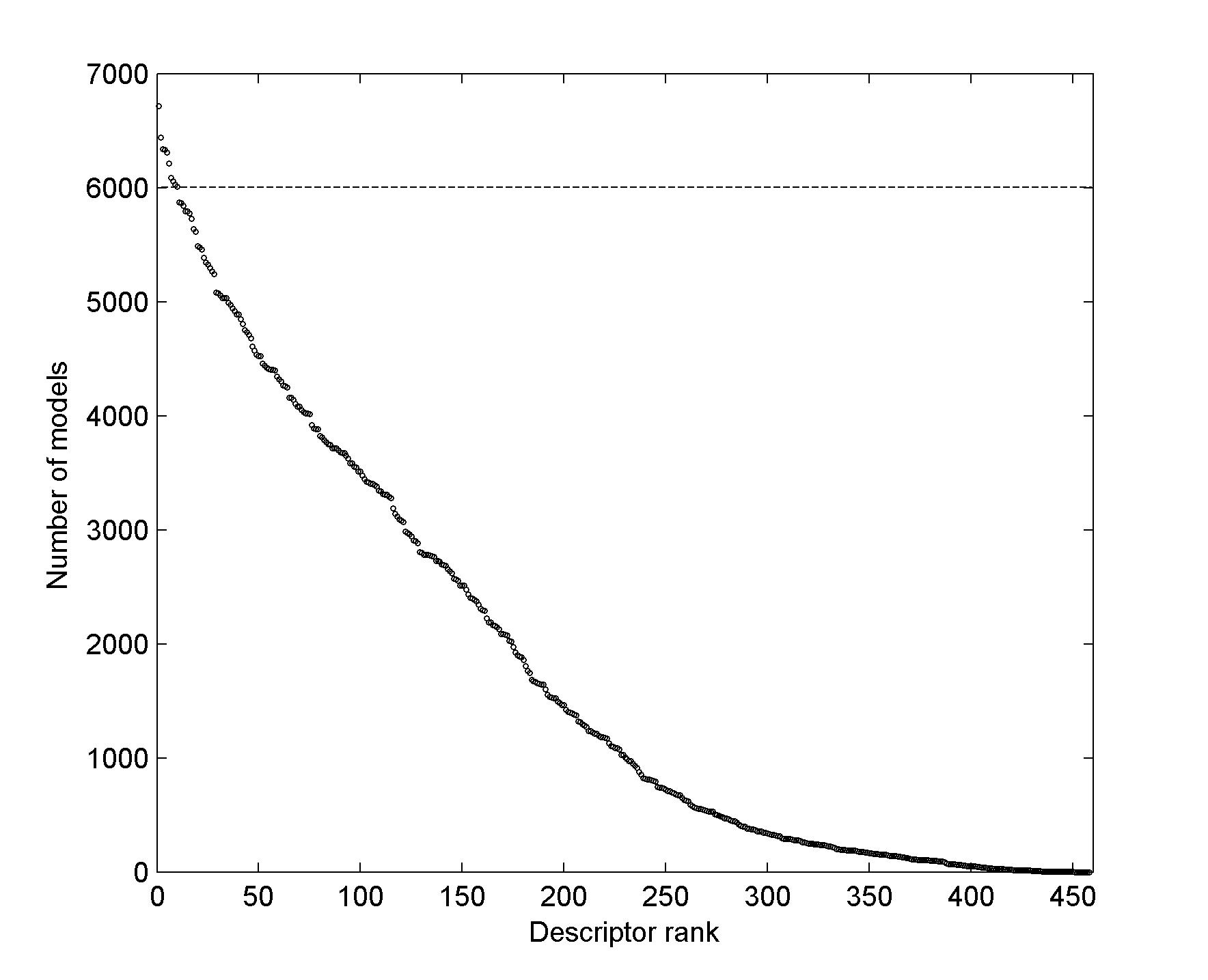


**Figure S3**. Frequency of the Mold2 descriptors used by the 2-class DF models


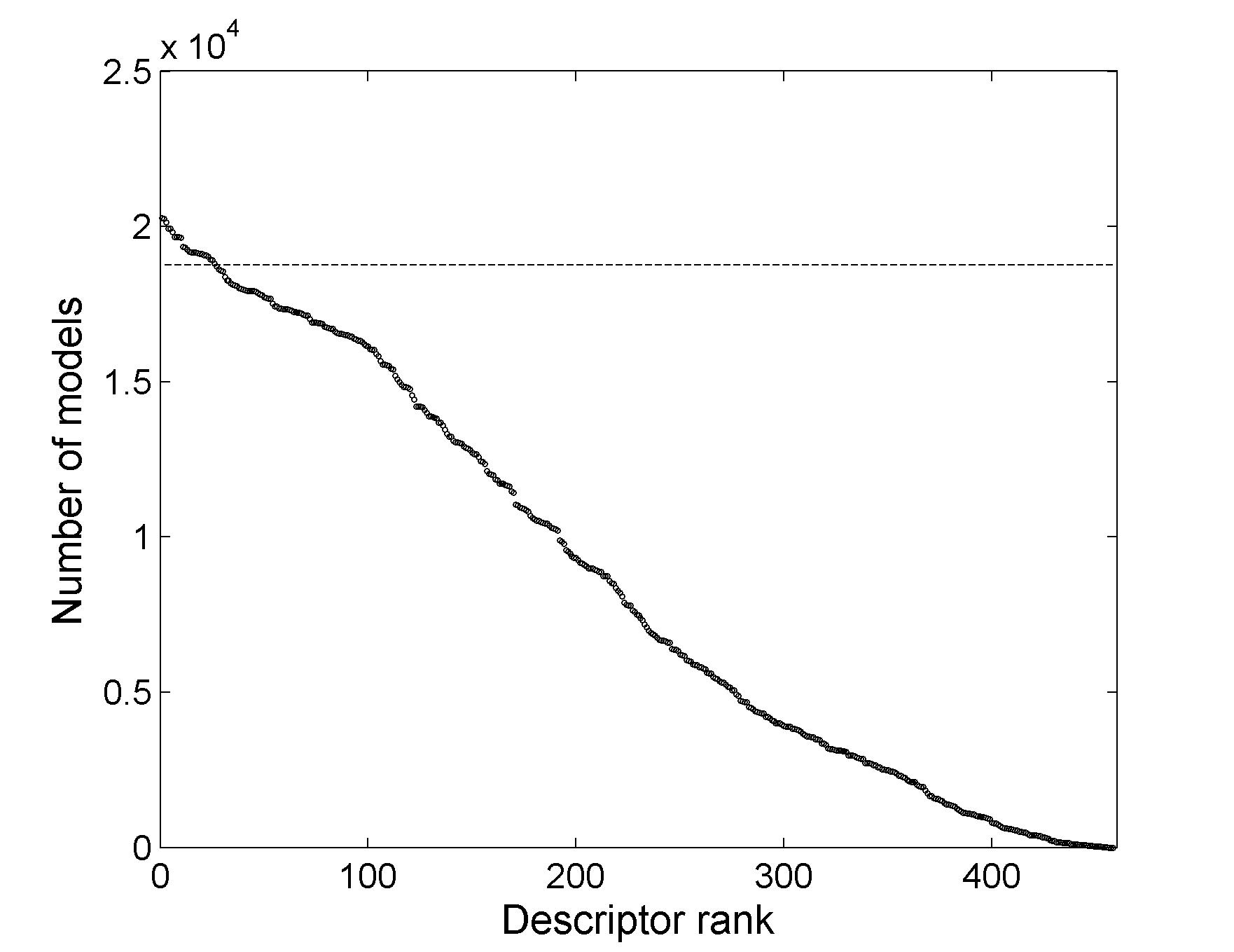


**Figure S4**. Frequency of the Mold2 descriptors used by the 3-class DF models


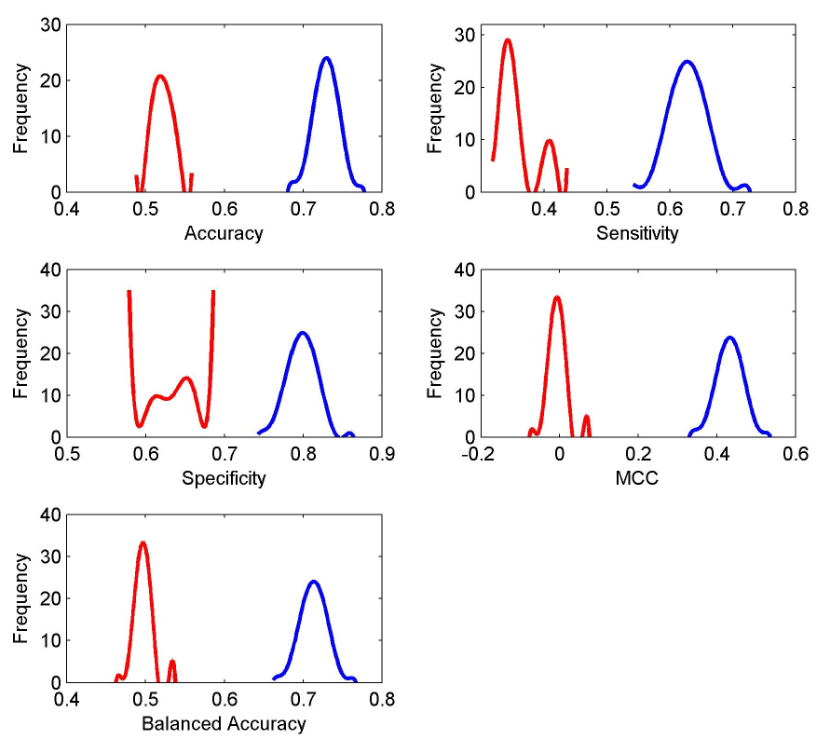


**Figure S5**. Comparison of the performance metrics (overall prediction accuracy, sensitivity, specificity, MCC, and balanced accuracy) of the 2-class prediction models between the 5-fold cross-validations (blue) and the permutation tests (red).


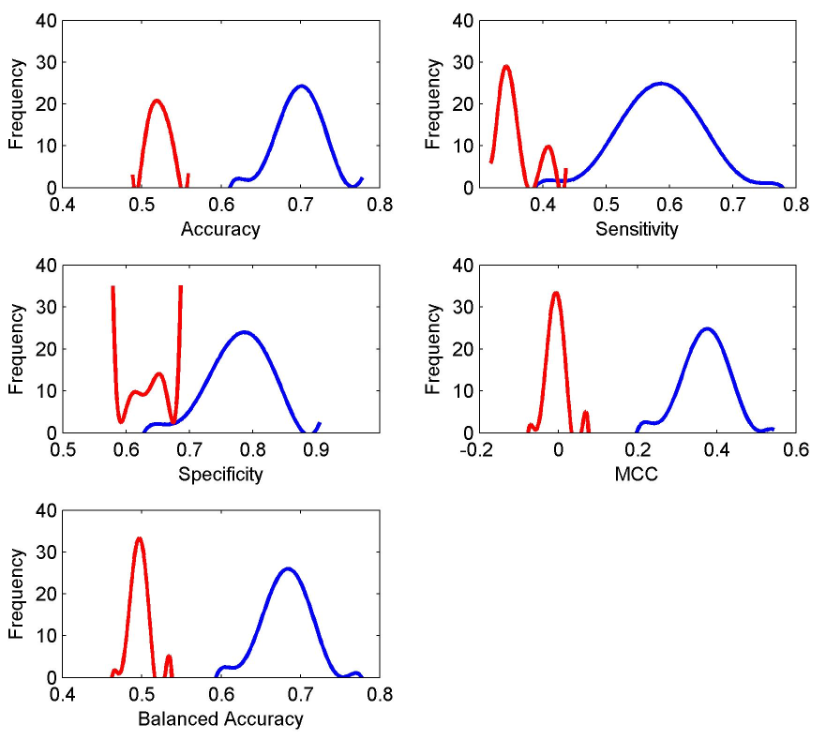


**Figure S6**. Comparison of the performance metrics (overall prediction accuracy, sensitivity, specificity, MCC, and balanced accuracy) of the 2-class prediction models between the bootstrapping (strategy A) validations (blue) and the permutation tests (red).


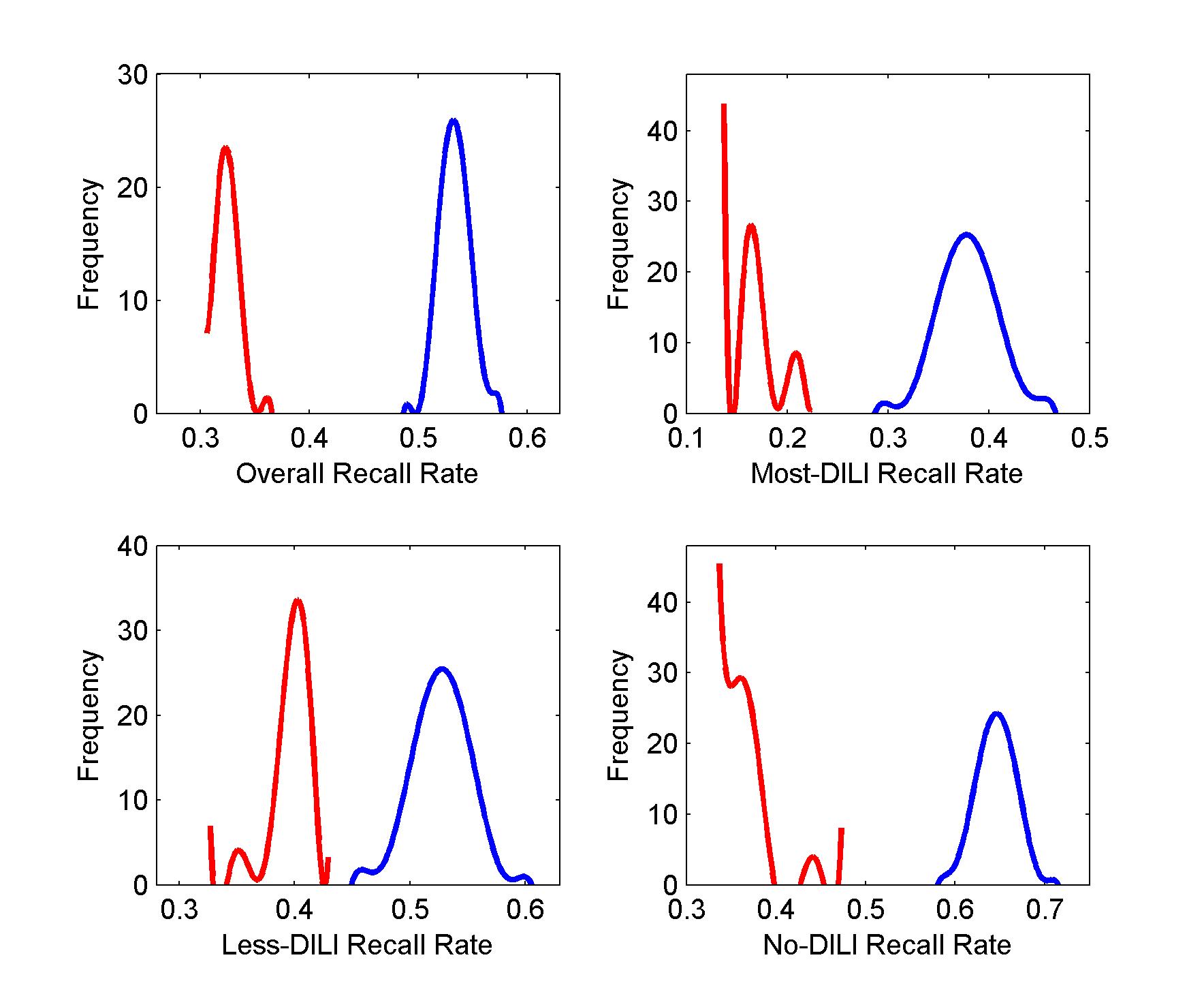


**Figure S7**. Comparison of the performance metrics (recall rates) of the 3-class prediction models between the 5-fold cross-validations (blue) and the permutation tests (red).


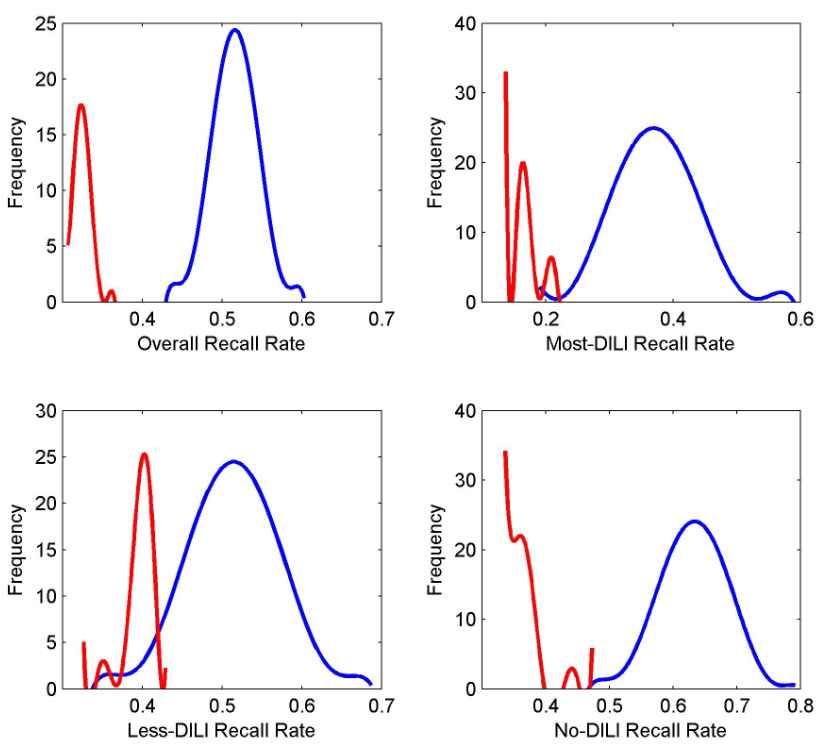


**Figure S8**. Comparison of the performance metrics (recall rates) of the 3-class prediction models between the bootstrapping (strategy A) validations (blue) and the permutation tests (red).


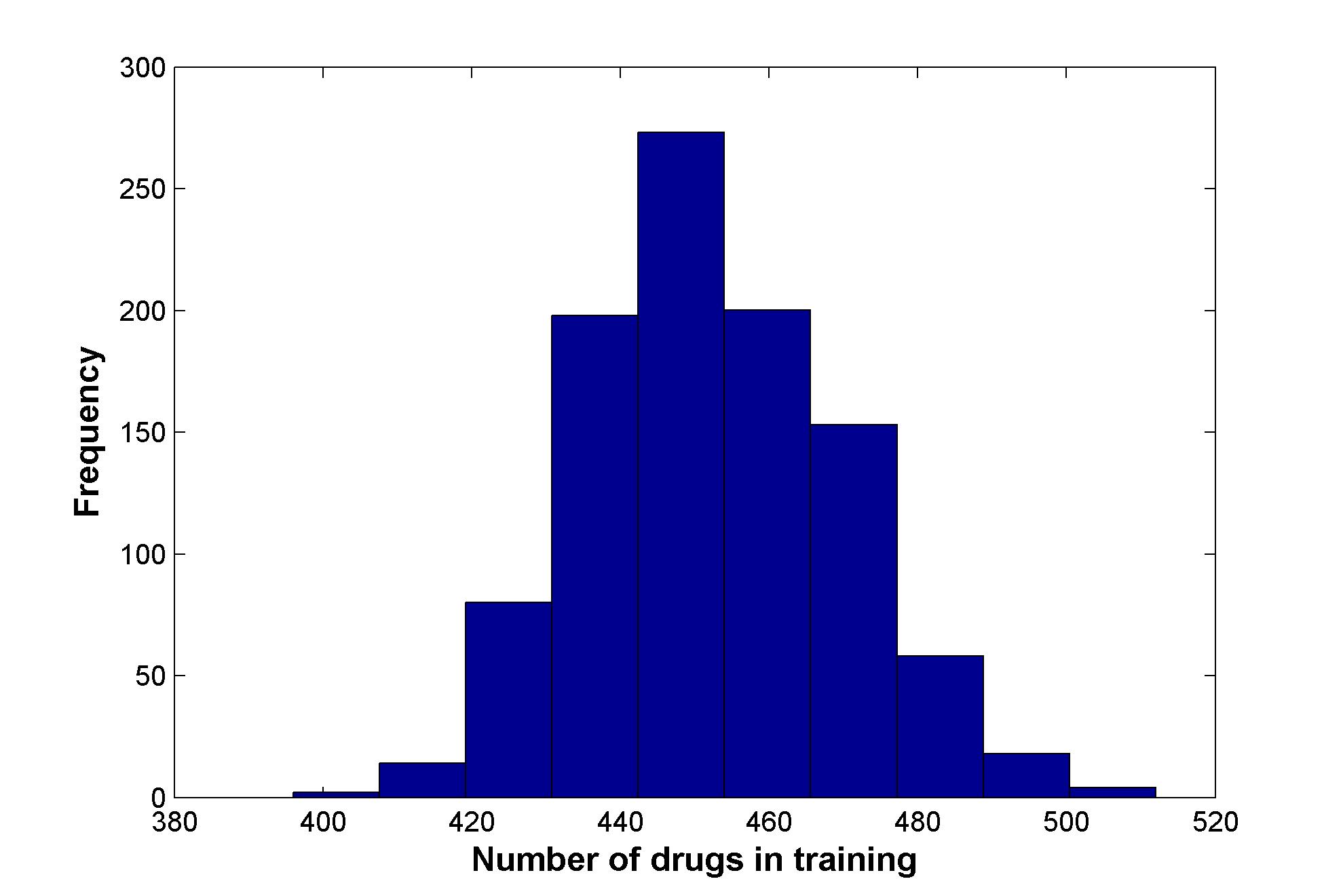


**Figure S9**. Distribution of the boot sizes (452±17, range between 396 and 512) from the 1000 iterations of bootstrapping (strategy A) for 2-class models.


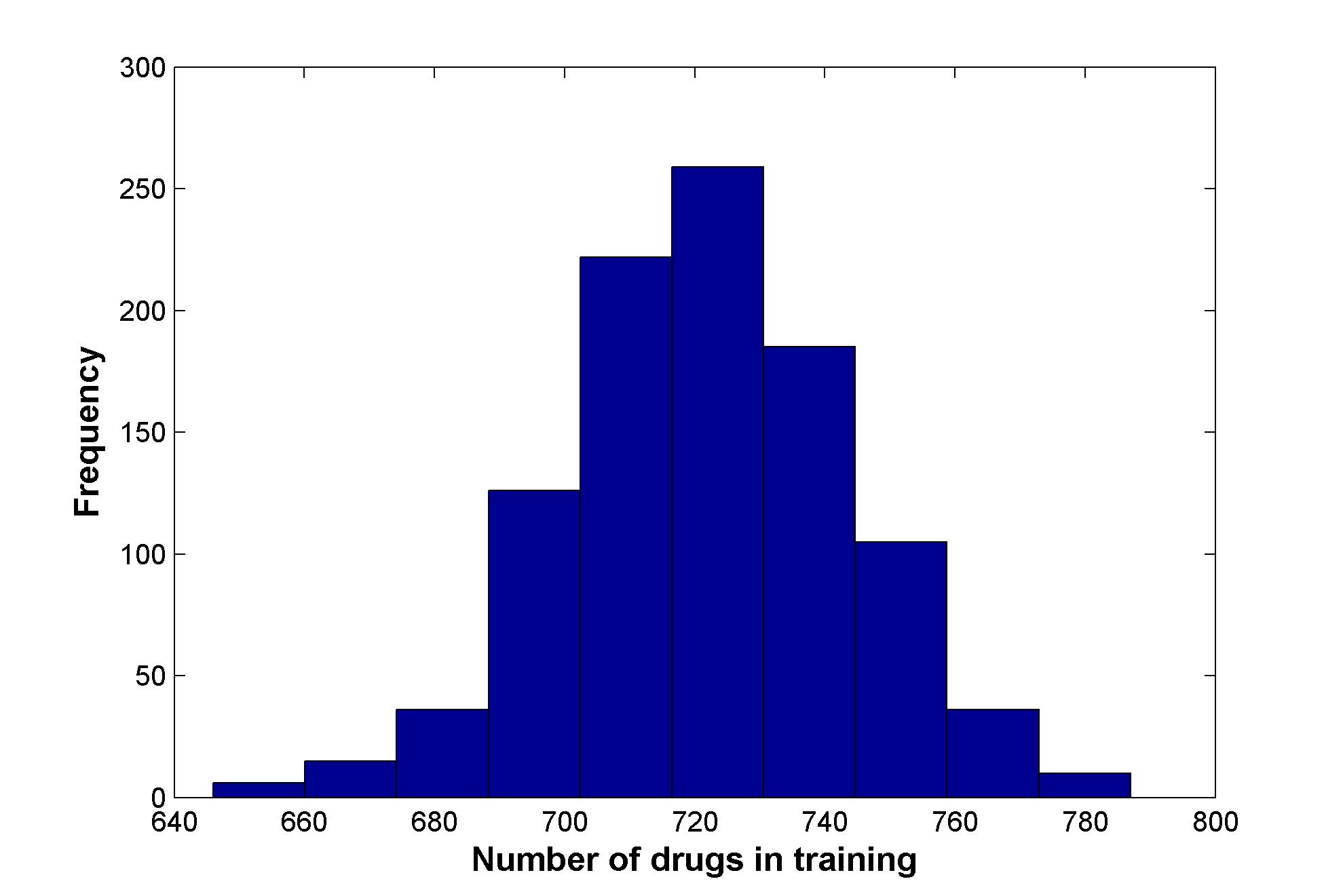


**Figure S10**. Distribution of the boot sizes (722±22, range between 646 and 787) from the 1000 iterations of bootstrapping (strategy A) for 3-class models.


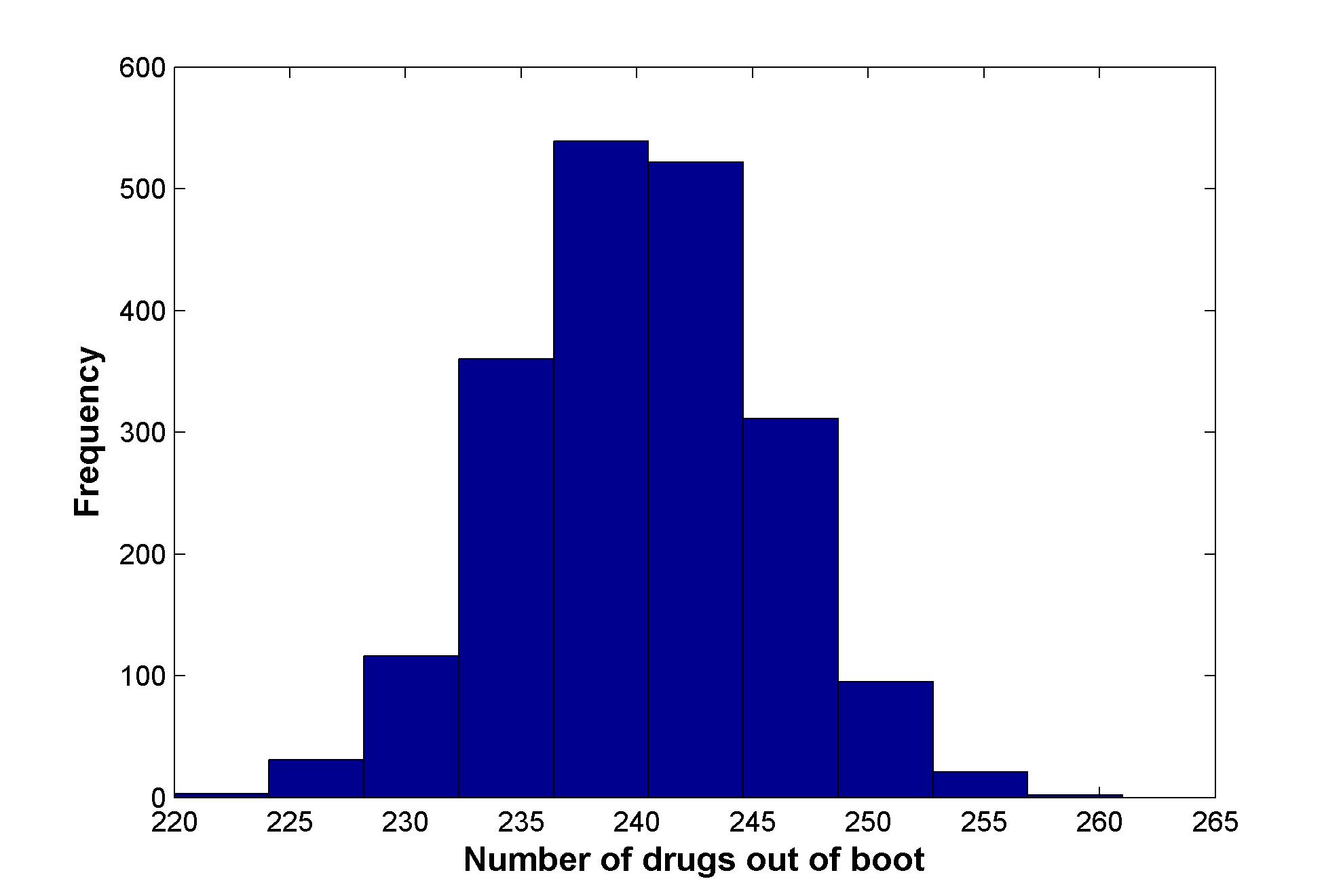


**Figure S11**. Distribution of the numbers of drugs out of boot (240±5, range between 220 and 261) from the 2000 iterations of bootstrapping (strategy B) for 2-class models.


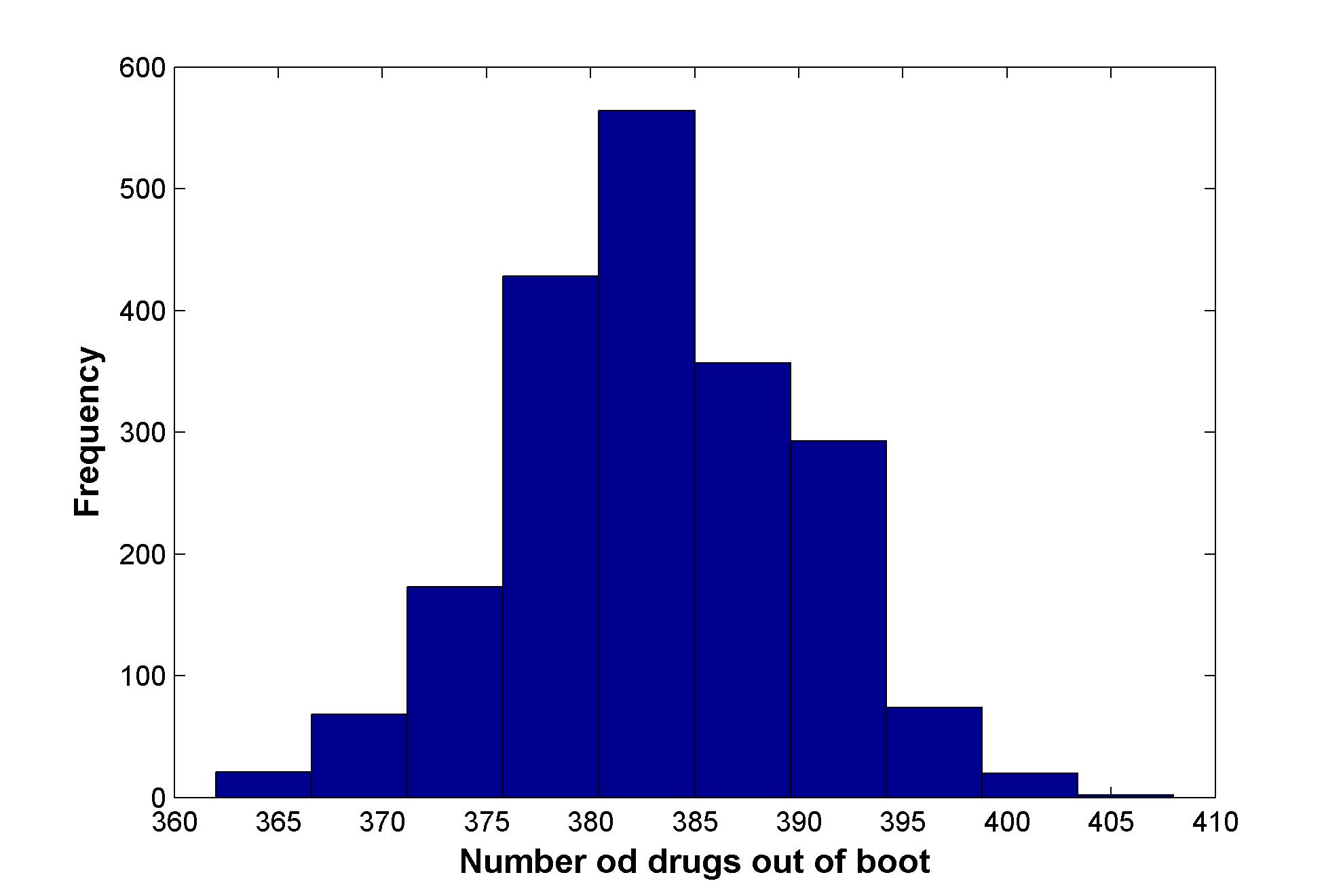


**Figure S12**. Distribution of the numbers of drugs out of boot (383±7, range between 362 and 408) from the 2000 iterations of bootstrapping (strategy B) for 3-class models.
